# 2-Pentadecyl-2-oxazoline ameliorates memory impairment and depression-like behaviour in neuropathic mice: possible role of adrenergic alpha2- and H3 histamine autoreceptors

**DOI:** 10.1186/s13041-020-00724-z

**Published:** 2021-02-08

**Authors:** Serena Boccella, Francesca Guida, Monica Iannotta, Fabio Arturo Iannotti, Rosmara Infantino, Flavia Ricciardi, Claudia Cristiano, Rosa Maria Vitale, Pietro Amodeo, Ida Marabese, Carmela Belardo, Vito de Novellis, Salvatore Paino, Enza Palazzo, Antonio Calignano, Vincenzo Di Marzo, Sabatino Maione, Livio Luongo

**Affiliations:** 1 Department of Experimental Medicine, Pharmacology Division, University of Campania “L. Vanvitelli”, 80138 Naples, Italy; 2 Department of Pharmacy, School of Medicine, University of Naples Federico II, Naples, Italy; 3 Institute of Biomolecular Chemistry, CNR, Pozzuoli, Italy; 4 Endocannabinoid Research Group, Institute of Biomolecular Chemistry, CNR, Pozzuoli, Italy; 5 Canada Excellence Research Chair on the Microbiome-Endocannabinoidome Axis in Metabolic Health, Université Laval, Quebec City, Canada; 6 IRCSS, Neuromed, Pozzilli, Italy

**Keywords:** Neuropathic pain, Depression, Cognitive impairments, H3 receptors, Locus coeruleus, Mice

## Abstract

Neuropathic pain (NP) remains an untreatable disease due to the complex pathophysiology that involves the whole pain neuraxis including the forebrain. Sensory dysfunctions such as allodynia and hyperalgesia are only part of the symptoms associated with neuropathic pain that extend to memory and affectivity deficits. The development of multi-target molecules might be a promising therapeutic strategy against the symptoms associated with NP. 2-pentadecyl-2-oxazoline (PEA-OXA) is a plant-derived agent, which has shown effectiveness against chronic pain and associated neuropsychiatric disorders. The molecular mechanisms by which PEA-OXA exerts its effects are, however, only partially known. In the current study, we show that PEA-OXA, besides being an alpha2 adrenergic receptor antagonist, also acts as a modulator at histamine H3 receptors, and report data on its effects on sensory, affective and cognitive symptoms associated with the spared nerve injury (SNI) model of neuropathic pain in mice. Treatment for 14 days with PEA-OXA after the onset of the symptoms associated with neuropathic pain resulted in the following effects: (i) allodynia was decreased; (ii) affective/cognitive impairment associated with SNI (depression, spatial, and working memories) was counteracted; (iii) long-term potentiation in vivo in the lateral entorhinal cortex-dentate gyrus (perforant pathway, LPP) was ameliorated, (iv) hippocampal glutamate, GABA, histamine, norepinephrine and dopamine level alterations after peripheral nerve injury were reversed, (v) expression level of the TH positive neurons in the Locus Coeruleus were normalized. Thus, a 16-day treatment with PEA-OXA alleviates the sensory, emotional, cognitive, electrophysiological and neurochemical alterations associated with SNI-induced neuropathic pain.

## Introduction

In the last two decades, many investigations have been focused towards plastic changes at supra-spinal cortical [1–3] and non-cortical structures [4, 5] underlying cognitive/affective consequences of neuropathic pain. Although the influence of neuropathic pain on hippocampal structures began to be studied many decades ago [6], only recently it has become the subject of more in-depth studies by several laboratories [7–10] The involvement of the hippocampus in spatial memory disorders and other comorbidities of neuropathic pain was recently shown also in the spared nerve injury (SNI) model of neuropathic pain [7]. SNI impairs synaptic plasticity (LTP or STP) at hippocampal dentate gyrus (DG), CA1, and CA3 synapses [7–10], as well as DG neurogenesis [7, 11]. The entorhinal cortex-hippocampus pathway plays a pivotal role in persistent pain such that cortical projections to the hippocampus can differently affect pain perception [12, 13] The interactions between the lateral entorhinal cortex (LEC) and the DG are in charge with the integration of important emotional stimuli associated with space–time orientation and place memory storage [14]. Among the several neurotransmitters and neuromodulators that orchestrate the complex scenario in forebrain chronic pain mechanisms, the glutamatergic (Glu), GABAergic and the endocannabinoid (eCB) systems have been deeply investigated [2, 15–18]. Furthermore, a key role of the biogenic amines (i.e. norepinephrine, dopamine, serotonin, histamine, etc.) has been highlighted in both chronic pain and some of its maladaptive neuroplasticities [19]. The noradrenergic projections modulating pain originate from cell nuclei in the brainstem, namely the locus coeruleus (LC), A5, and A7 cell groups [20, 21]. In particular, the noradrenergic system in the LC seems to play a key role in the maintenance of neuropathic pain-induced allodynia [22]. Like norepinephrine, histamine is gaining great interest in the central mechanisms of neuroplasticity underlying neuropathic pain [23, 24]. In addition to the known sedative effect of histamine H1 receptor antagonists, interest today has focused on histamine H3 receptors [24–26], which, by being expressed at the level of the presynaptic terminals, strongly influence the release of histamine and various neurotransmitters [27–29]. Although histamine H3 receptor blockade has been shown to relieve neuropathic pain [25, 26], the role of this receptor and the mechanisms underlying its supraspinal effects on the various components of neuropathic pain are still poorly understood. We recently investigated the behavioral effects of 2-pentadecyl-2-oxazoline (PEA-OXA), a natural compound found in green and roasted coffee beans, in a murine model of mild traumatic brain injury (mTBI), which develops into a neuro-psychiatric syndrome very often observed in post-traumatic states. In that study, it was found that the in vitro and in vivo effects of PEA-OXA were mediated by its action as an antagonist on the norepinephrine alpha2 receptor [30]. The evidence that the analgesic effects of H3 receptor antagonists/inverse agonists in neuropathic pain can be partially mediated by α2 adrenoceptor desensitization [23] inspired us to evaluate possible anti-neuropathic properties of PEA-OXA and investigate the possible involvement of H3 receptor.

In the present study, we first checked whether PEA-OXA was also able to modulate the activity of histamine H3 receptors in vitro. Indeed, this compound belongs to a large family of long chain fatty acid-derived biologically active mediators with multiple molecular targets [31]. Once that its pharmacological activity at the histamine H3 receptor was demonstrated for the first time, we evaluated the effect of repeated administrations (16 days starting the treatment 14 days after sciatic nerve surgery) of PEA-OXA on: (i) mechanical allodynia, (ii) affective and cognitive (spatial and working memory) behavior, (iii) neural activity in the LC, (iv) in vivo long term potentiation in the entorhinal-cortex/dentate gyrus, LPP, and (iv) glutamate, GABA, norepinephrine, dopamine and histamine levels in the hippocampus. All the behavioral, biochemical and electrophysiological experiments were carried out in control and neuropathic mice 30 days after the sham or SNI surgery on the sciatic nerve.

## Materials and methods

### *Homology modelling of histamine H*_*3*_* receptor*

The sequences of the histamine H_3_ receptor was retrieved from the publicly available sequence database www.uniprot.org (UNIPROT: Q9Y5N1). 50 homology models of receptor were built with MODELLER [32, 33] version 9.14 using the x-ray structure of the acetylcholine M_3_ receptor (PDB id: 4U15) as a template and the structural alignment. The best model in term of both Modeller Objective Function and Dope score was selected for the subsequent docking calculations.

**Fig. 1:**
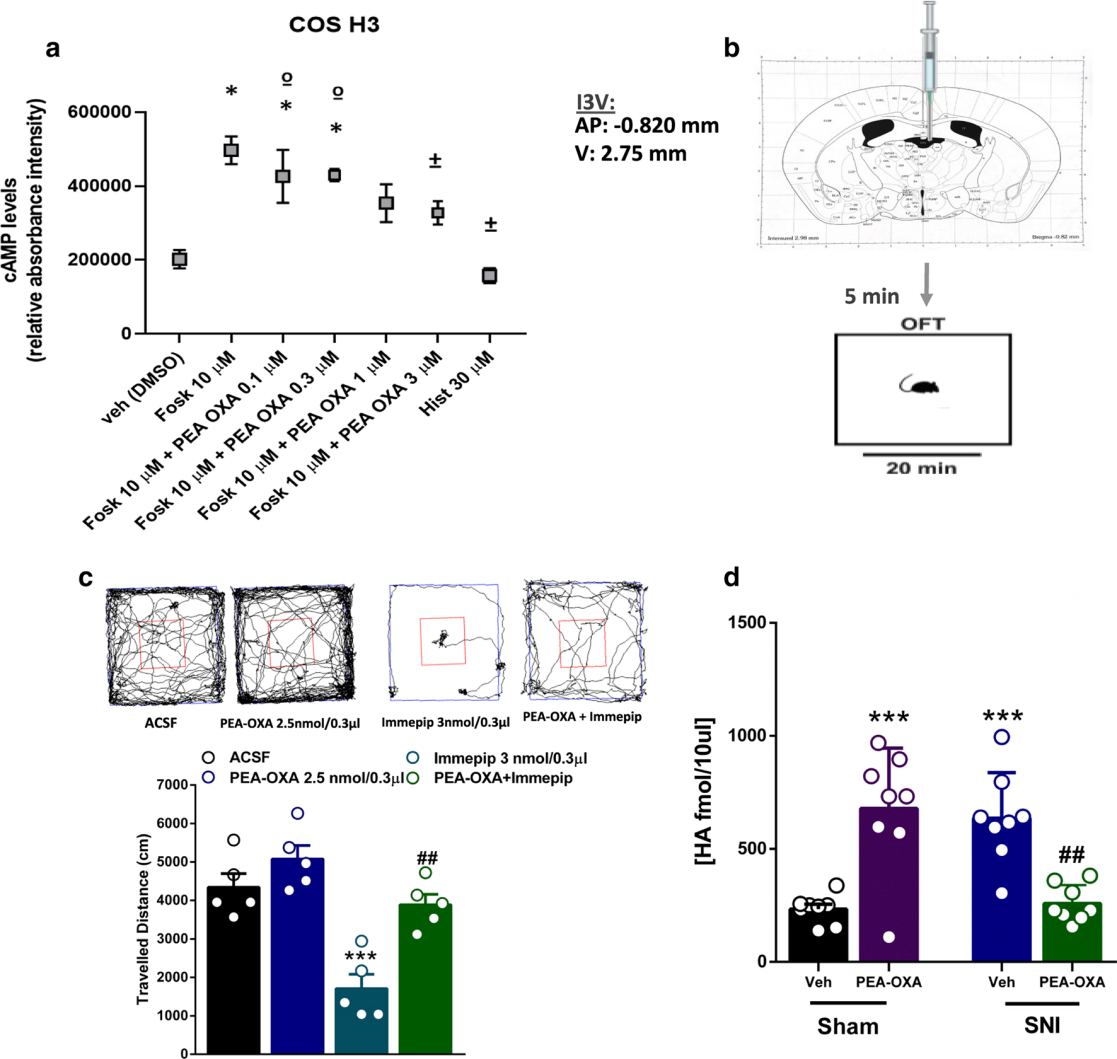
Effect of PEA-OXA in COS cells stably expressing human histamine H3 receptors (**a**). Scatter plots showing the effect of PEA-OXA in COS cells expressing histamine H3 receptors on intracellular cAMP levels. Data represent the mean ± S.E.M. of four separate determinations. Data sets were compared using t-Test and ANOVA followed by Tukey’s test. The asterisk indicates a p value ≤ 0.05 vs vehicle. The symbol (±) indicates a p value ≤ 0.05 vs forskolin. The symbol (º) indicates a p value ≤ 0.05 vs histamine. Effects of single injection of PEA-OXA (2.5 nmol/0.3 μl i.c.v.) on Immepip-induced decreased locomotor activity in into third ventricle (I3V) of Naϊve mice (**b**, **c**). Representation of coronal sections of the mouse brain with the cannula placement in I3V (**b**), Representative traces of mouse movement during an open field test (**c** upper panel). Total distance traveled in OFT (**c** lower panel). Data are represented as mean ± SEM of 5 mice per group. **p < 0.01 and ^###^p < 0.001 indicate significant differences compared to ACSF or Immepip. One-Way ANOVA, followed by Holm-Sidak's post hoc test for multiple comparisons test was performed. Effect of the chronic treatment with vehicle (kolliphor 5% in saline, v/v, i.p.) or PEA-OXA (10 mg/kg, i.p.) on the Histamine release in the hippocampus CA3 (**d**). Data are represented as mean ± SEM of 8 mice per group. Two-way ANOVA, followed by Tukey's post hoc test for multiple comparisons test were used for statistical analysis. p < 0.05 was considered statistically significant. Symbols indicate significant differences: ***vs Sham/veh (p < 0.0001) and ^##^vs SNI/veh (p < 0.001), respectively

### Ligand building

To carry out the docking calculations, the starting ligand geometry of histamine and PEA-OXA was built with UCSF Chimera 1.14 [34] and energy minimized at molecular mechanics level, using AM1-BCC charges and then optimized using GAMESS program [35] at the Hartree–Fock level with STO-3G basis set, followed by a single-point HF energy evaluation at the 6-31G* level to derive the partial atomic charges for the ligands using the RESP procedure [36].

### Molecular docking

Docking studies were performed with AutoDock 4.2 [36, 37]. H_3_ model along with the ligands was processed with AutoDock Tools (ADT) package version 1.5.6rc123 to merge non-polar hydrogens, calculate Gasteiger charges and select rotatable sidechain bonds. A 60 Å × 60 Å × 70 Å grid, centred in the orthosteric binding pocket, was generated with the program AutoGrid 4.2 included in Autodock 4.2 distribution, with a spacing of 0.375 Å. Docking runs were carried out by either keeping fixed the whole protein or alternately allowing the rotation of triples of selected residues (Asp114/Glu206/Trp402 for both histamine and PEA-OXA, Tyr374/Tyr394/Phe398 and Tyr374/Thr375/Ser203 for pea-oxa alone). 100 molecular AutoDock docking runs for each docking calculation were performed adopting a Lamarckian Genetic Algorithm (LGA) and protocol already published [38]. Flexibility was used for all rotatable bonds of both docked ligands. The representative poses for each ligand were selected based on binding energy value for the subsequent energy minimization with Amber16 [39] package using ff14SB version of AMBER ff14SB force field for the protein and gaff parameters for the ligand. UCSF Chimera 1.14 was used for figures of molecular complexes.

### Cell culture, reagents ad transfection

Fibroblast-like cells (COS-7; ATCC CRL-1651) were plated onto 24-mm plastic Petri dishes in Dulbecco’s modified Eagle’s medium (DMEM) containing 10% FBS, nonessential amino acids (0.1 mM), penicillin (50 U/ml), and streptomycin (100 µg/ml) in a humidified atmosphere at 95% O_2_/5% CO_2_ at 37 °C. The next day, the cells were transfected with the following plasmids: (i) human histamine H1 receptor (Origene Technologies, MD, USA; Cat. RG209217); (ii) human histamine H3 receptor (Origene Technologies, MD, USA; Cat. RG218388) and (iii) human histamine H4 receptor (Origene Technologies, MD, USA; Cat. RG212974) using Lipofectamine 2000 (Thermo Fisher, Milan, IT: Cat. 11668027). A negative scramble control vector (Origene Technologies, MD, USA; Cat. SKUGE100003) was used as control. After 24 h, the cell culture media was replaced with fresh DMEM containing G418 (Geneticin) antibiotic (Thermo Fisher, Milan, IT: Cat. 10131027). The stable clones were selected adding 0.6 µg/ml G418 in the media for one month. Cell clones were selected by quantitative PCR.

### RNA purification and quantitative real-time PCR (qPCR)

Total RNA was isolated from cells using Pure Link^®^ RNA Mini Kit (Cat. N.: 12183018A; Thermo Fisher Scientific, Milan, Italy) following manufacturer’s instruction, and then quantified by spectrophotometric analysis. The purified mRNA was reverse-transcribed by use of iScript reverse transcriptase enzyme (Cat. N.: 1708840; Biorad, Milan, Italy). Quantitative real-time PCR was carried out in CFX384 real-time PCR detection system (Bio-Rad, Milan, Italy) with specific primers: H1 forward: CTGAGCACTATCTGCTTGGTC, H1 reverse: AGGATGTTCATAGGCATGACGA; H3 forward: GCCACTGCTATGCCGAGTT, H3 reverse: TGCGCCTCTGGATGTTCAG; H4 forward: ATGCTAGGAAATGCTTTGGTCA,H4 reverse: AGGAATGGAGATCACACCCAC; S16 forward: TCGGACGCAAGAAGACAGCGA, S16 reverse: AGCGTGCGCGGCTCAATCAT using ssoAdvance Universal SYBR Green Supermix (Cat. N.: 1725270 Bio-Rad, Milan, Italy). Samples were amplified simultaneously in quadruplicate in a one-assay run with a non-template control blank for each primer pair to control for contamination or primer-dimers formation, and the ct (cycle threshold) value for each experimental group was determined. The housekeeping genes (the ribosomal protein S16) have been used as an internal control to normalize the ct values using the 2^−*∆∆ct*^ formula [40].

### Cell viability assay

H3 or H4 COS cells were plated onto 24 multi-well Petri dishes at the confluence of 60–70%. The day next were treated with a increasing concentration of PEA-OXA (up to 10 µM) for 24 h. Dimethylsulfoxide (DMSO) was used as the vehicle. Cell viability was assessed by MTT (3-(4,5-dimethylthiazol-2-yl)-2,5-diphenyltetrazolium bromide; Cat. M2003 Sigma Aldrich) assay following published procedures [41].

### Measurement of cAMP

Adenosine-3′, 5′-cyclic monophosphate or cyclic AMP (cAMP) was measured in control and H3 or H4 transfected COS cells using the direct cyclic AMP immunoassay kit (Arbor Assays, Michigan, USA) following manufacturer's instructions.

### Intracellular calcium [Ca^2+^]_i_ measurement

The intracellular [Ca^2+^]_i_ measurement was performed in H1R transfected COS cells according to published procedures [40].

### Animals

4–5 weeks old male C57BL/6J mice (Harlan, Italy) weighing 18–20 g were housed three per cage under controlled illumination (12 h light/dark cycle; light on 6:00 A.M.) and standard environmental conditions (ambient temperature 20–22 °C, humidity 55–60%) for at least 1 week before the commencement of experiments. During this period, mice were accustomed to handling in order to minimize stress. Mice chow and tap water were available ad libitum. All experiments were carried out during the light cycle. The experimental procedures were approved by the Animal Ethics Committee of University of Campania “L. Vanvitelli” of Naples. Animal care was in compliance with Italian (D.L. 116/92) and European Commission (O.J. of E.C. L358/1 18/12/86) regulations on the protection of laboratory animals. Mice were assigned randomly in four experimental groups identified by an alphanumeric code. Experimenter was blind to the treatments. All efforts were made to reduce both animal numbers and suffering during the experiments. For in vivo experiments were used a total number of 120 animals and the exact number of animals used for each item can be seen both in plotted graphs or results. Behavioral tasks were scheduled in order to avoid carry-over effects from prior testing experience and each experimental group was divided in several subgroups of the same age.

### Neuropathic pain induction

The spared nerve injury of the sciatic nerve was performed according to Decosterd and Woolf [42]. The mice were first anaesthetized by tribromoethanol (250 mg/kg, i.p.) after which the sciatic nerve was exposed at the level of its trifurcation in sural, tibial and common peroneal nerves. The tibial and common peroneal nerves were ligated tightly with 7.0 silk threads and then transected just distal to the ligation, leaving the sural nerve intact. Sham mice were anaesthetized, the sciatic nerve was exposed at the same level, but it was not transected. The behavioral, electrophysiological and microdialysis experiments have been carried out 30 days from the SNI or sham surgery (day 0). Experimenter was blind to the treatments.

### Tactile allodynia

Mechanical allodynia was measured with a series of calibrated von Frey nylon filaments (Stoelting, Wood Dale, IL, USA), ranging from 0.008 to 2 g. The neuropathic and sham mice were left free to move in the enclosure compartment positioned on the surface of the wire mesh for about 1 h before the test. The von Frey filaments were applied in increasing order on the mid-plantar surface of the hind paw through the mesh floor. If the use of the filament three times did not induce a reaction, the next filament with higher pressure was used. The behavioral responses (rapid withdrawal of the paw, licking or shaking of the paw, during the application or immediately after the removal of the filament) were expressed as the mean ± S.E.M. of the paw withdrawal threshold (PWT) in grams. Tactile allodynia (PWT) was measured on day 0 (baseline) and on the 14th day after sham or SNI surgery when allodynia was fully developed. On the 14th day, the treatment with PEA-OXA (10 mg/kg i.p.) or vehicle (kolliphor 5% in saline solution) also started. Tactile allodynia was measured 7 and 16 days after the start of treatment, corresponding to the 21st and 30th day (D21, D30) from the SNI or sham surgery.

### Depression-like behaviour

#### Forced swimming test

Mice were individually placed in a cylinder (30 cm × 45 cm) filled with water at a temperature of 27 °C, for a 6-min period. The duration of immobility, expressed in seconds, was monitored during the last 4-min of the 6-min session, because little immobility is observed during the first 2 min. Immobility was defined as the absence of escape-oriented behaviour (floating in the water without struggling and making only those movements necessary to keep head above the water). Immediately after the test, the mice, taken out from the swimming pool, were put to dry under a heating lamp [43].

#### Tail suspension

The mice were individually suspended from the tail on a horizontal bar 50 cm away from the floor using adhesive tape. The duration of the immobility, recorded in seconds, was monitored during the last 4 min of the 6 min-test by a time recorder. Immobility was considered to be the absence of any escape-oriented movement [43].

### Anxiety-like behaviour

#### Elevated plus-maze

The apparatus consisted of two opposite open and closed arms (35 cm × 7 cm) elevated 50 cm from the floor. Each mouse was placed in the central square of the maze (5 × 5 cm), facing one of the closed arms and allowed to move freely for 5 min. The open-arm choice was calculated as the ratio of open-arm entries to the total number of entries and expressed as %. It was recorded using a camera capture and analyzed using a video tracking software (Any-maze, Stoelting, Wood Dale, IL, USA) [44].

#### Light–dark box

The light–dark box apparatus consisted of two equally sized compartments (60 cm × 30 cm × 30 cm; length × width × height): the dark compartment (black perspex) was covered, whereas the light compartment (white perspex) was open and brightly lit from above (~ 150 lx). Access between compartments was allowed through a partition door (70 × 70 mm). At the beginning of the session, mice were placed in the light compartment and let free to explore for 10 min. The time spent in the dark compartment was recorded [43].

#### Marble burying

Mice were individually placed in a transparent cage (42 cm × 24 cm × 12 cm length × width × height) containing 5 cm layer of sawdust bedding and 15 glass marbles (1.5 cm in diameter) arranged in three rows. Mice were left undisturbed for 15 min under dim light. An observer blind to the treatment counted the number of digging and marbles buried (at least two or third buried in the sawdust). At the end of the test, the mouse was removed to its cage [44]

### Cognitive performance

#### Morris water maze

Morris water maze apparatus consists of a circular water tank (diameter 170 cm, height 60 cm) filled with water (24 ± 1 °C) containing an invisible plexiglass platform (10 cm in diameter) submerged under (1.5 cm below) the surface of the water. Swimming was recorded using a camera capture and analyzed using a video tracking software (Any-maze, Stoelting, Wood Dale, IL, USA) that divided the pool into four equal quadrants: NE, SE, SW, and NW. The escape platform was placed in the midpoint of the SW and the position remained stable for 5 days. Mice were trained daily for four trials per day for 5 days, with an inter-trial interval of 15 min and the start position was pseudorandomized across trials. Each trial was started by placing a mouse into the pool, facing the wall of the tank and terminated as soon as the animal reaches the platform with a cut-off of 60 s. Average of the four trials for each mouse of each group was expressed as mean ± SEM of the time of latency to reach the platform in seconds for each training day. A probe test was performed 1 h after the last swim on day 5. The platform was removed from the tank and each mouse was allowed to freely swim for 60 s. The time spent in the quadrant where the platform was previously placed, was determined and expressed in seconds and interpreted as a higher level of memory retention. Following the probe trial, reversal training was conducted for a further 3 days. During reversal training, the escape platform was moved to the midpoint of the opposite quadrant (NE) and mice were let free to swim for four trials per day. A second probe trial was performed 1 h after the last swim (day 8) [7].

#### Y-maze

The apparatus consisted of three enclosed arms (30 × 5 × 15 cm; length × width × height) converging on an equilateral triangular center (5 × 5 × 5 cm). At the beginning of each experimental session, each mouse was placed in the center platform and the number of spontaneous alternations (defined as the number of successive triplet entry into each of the three arms without any repeated entry) was monitored in a 5 min test session. The percentage of alternation was calculated as the percentage of the ratio of the number of alternations/(total number of arm entries − 2) × 100.

#### Open field test

Open field test has been set as described by [45]. This test has been performed only in Naïve mice in order to demonstrate a H3-mediated mechanism. The naϊve mice were implanted with a further cannula into the third ventricle (I3V, AP: − 0.820 mm; V: 2.75 mm) for microinjecting of Immepip 3 nmol/0.3 µl or PEA-OXA 2.5 nmol/0.3 µl. Immepip were administrated for 3 days before the open field test. Behavioral assays were performed 5 min after drugs injection. The apparatus was cleaned before each behavioral session by solution of 70% EtOH. Naϊve mice were randomly assigned to a treatment group. Behaviors were recorded, stored, and analyzed using an automated behavioral tracking system (Smart v3.0, Panlab Harvard Apparatus). Mice were placed in an OFT arena (l × w × h: 25 cm × 25 cm), and ambulatory activity (total distance travelled in centimeter), were recorded for 20 min and analyzed.

### In vivo microdialysis

Mice were anaesthetized (pentobarbital, 50 mg/kg, i.p.) and stereotaxically implanted with concentric microdialysis probes into the CA3 (AP: − 2.46 mm from bregma, L: 2.75 mm from midline and V: 2.6 mm below dura or dentate gyrus (DG, AP: − 1.7 mm from bregma, L: 1 mm from midline and V: 1.8 mm below dura) areas of the hippocampus [46]. Microdialysis concentric probes were constructed as described by Hutson et al. [47] with 25G (0.3 mm inner diameter, 0.5 mm outer diameter, A-M Systems) stainless steel tubing and inlet and outlet cannulae (0.04 mm I.D., 0.14 mm O.D.) consisting of fused silica tubing (Scientific Glass Engineering, Melbourne, Australia). The microdialysis probe had a tubular dialysis membrane (Enka AG, Wuppertal, Germany) of 0.8 mm in length. After 24 h of recovery, in vivo microdialysis was performed in awake and freely moving mice by perfusing dialysis probes with artificial cerebrospinal fluid (CSF composition in mM: KCl, 2.5; NaCl, 125; MgCl_2_, 1.18; CaCl_2_, 1.26) (pH 7.2) at a rate of 1.0 μl/min using a Harvard Apparatus infusion pump (mod. 22). After a 60 min equilibration period, 6 consecutive 30-min dialysate samples were collected for detecting the basal release of neurotransmitters following PEA-OXA or vehicle chronic treatments. For single PEA-OXA or vehicle administration, 12 consecutive dialysate samples were collected (5 pre-administration and 7 post-administration. On completion of experiments, mice were anaesthetized with pentobarbital and their brains perfused–fixed via the left cardiac ventricle with heparinized paraformaldehyde saline (4%). Brains were dissected out and fixed in a 10% formaldehyde solution for 2 days. Each brain was cut in 40 μm thick slices and observed under a light microscope to identify the probe tip locations.

Dialysates were analyzed for amino acid content using high-performance liquid chromatography (HPLC) with the fluorimetric detection method. The system comprised a Varian ternary pump (mod. 9010), a C18 reverse-phase column, a Varian refrigerated autoinjector (mod. 9100) and a Varian fluorimetric detector. Dialysates were precolumn derivatized with *o*-pthaldialdehyde-*N*-acetylcysteine (OPA-NAC) (10 μl dialysate + 5 μl OPA-NAC + 10 μl borate buffer 10%) and amino acid conjugates re-solved using a gradient separation. The mobile phase consisted of two components: (1) 0.2 M sodium phosphates buffer and 0.1 M citric acid (pH 5.8) and (2) 90% acetonitrile and 10% distilled water. Gradient composition was determined using an Apple microcomputer installed with Gilson gradient management software. Data were collected using a Dell Corporation PC system 310 interfaced to the detector via a Drew data-collection unit. The mean dialysate concentration of amino acids in the six samples represented the basal release and the results were expressed as the mean ± SEM of the pmol in 10 μl of perfusate sample.

Biogenic amines (norepinephrine, dopamine and histamine) content was assessed by HPLC with the electrochemical detector method, consisting of a model 590 pump (Waters Associates, Milforal, USA) and an electrochemical detector BIORAD mod. 1640 with an Ag/AgCl reference electrode. A C-18 reverse-phase analytical column (Discovery, 5 µm, 4.6 mm i.d. × 150 mm; SUPELCO, USA) was eluted using different mobile phases depending on the amine and maintained at a fixed temperature of 40 °C. Mobile phase composition and flow rate were: 12.5% methanol, 0.15 mM NaH_2_PO_4_, 0.01 mM octanosulfonic acid, 0.5 mM EDTA (pH 3.8), flow rate 1 ml/min for dopamine; 12% methanol, 0.1 M sodium acetate, 0.3 mM EDTA, 1.8 mM octanosulfonic acid (pH 5.4), flow rate 0.8 ml/min for norepinephrine; and 20% methanol, 100 mM NaH_2_PO_4_, 5.2 mM octanosulfonic acid (pH 6), flow rate 0.8 ml/min for histamine. Detection potential was set at 0.55 V. Volume injection was 10 µl for dopamine and norepinephrine and 20 µl for histamine which requires derivatization with OPA-Na_2_SO_3_ solution [48] in a ratio of 10:2 (10 µl of dialysate and 2 µl of derivatization solution, and 8 µl of ACSF). OPA-Na_2_SO_3_ stock solution was composed of OPA 25 mM, sodium sulfite 125 mM, and 0.1 mM sodium borate buffer (pH 10.4). The mean concentration of norepinephrine, dopamine and histamine in the six dialysate samples represented the basal release and the results were expressed as the mean ± S.E.M of the fmol in 10 µl of perfusate. For the single PEA-OXA or vehicle administration the percentage of concentration variation after administration was considered.

### In vivo recording of long-term potentiation (LTP) in the LEC-DG pathway

Under urethane anaesthesia (1.5 g/kg, i.p) the mice were fixed on a stereotaxic apparatus (Stoelting Co USA) in order to insert a stimulation electrode in the angular bundle of the lateral entorhinal cortex (LEC) and a recording electrode in the dentate gyrus hilum (DG), following the coordinates of the atlas of Paxinos and Franklin [46]. The stimulating and recording electrodes were slowly lowered in the areas mentioned above until a field excitatory post-synaptic potential (fEPSP) appeared under low-frequency stimulation (0.033 Hz). A 30 min stable baseline was recorded followed by the delivery to the LEC of tetanus (TBS) consisting of six trains, six bursts, six pulses a 400 Hz, inter-burst interval: 200 ms, inter-train interval: 20 s. Evoked responses after TBS, were recorded for 90–120 min. LTP was considered if the amplitude and the slope of fEPSPs increased more than 20% for at least 30 min after the TBS [49, 50]. The fEPSPs recorded before and after LTP were stored for analysis of slope and spike amplitude (WinLTP 2.30, Bristol, UK). All data points were normalized to the average baseline and data were analyzed by using ANOVA.

### In vivo recordings of LC neuron activity

Mice were anaesthetized with chloral hydrate (400 mg/kg, i.p.) and fixed in a stereotaxic apparatus (David Kopf Instruments, Tujunga, CA, USA). The level of anaesthesia was measured by the absence of a reflex nociceptive reaction to a pinch of the tail or paw and an eye blink response to pressure. In order to maintain a full anaesthetic state during the experiments, supplemental doses of chloral hydrate (120 mg/kg, i.p.) were given when required. The skull was exposed and a hole drilled for the placement of a recording electrode (3–5 MΏ of impedance, FHC Frederick Haer & Co., ME) into the LC (AP: − 5.4 mm from bregma; L: 0.4 to 1 mm from midline and V: 2.7–4 mm below the dura) [46] contralaterally to the hind paw which has undergone the sham or SNI surgery. Single-unit extracellular recordings of LC neurons were performed 30 days after surgery.

The activity of noradrenergic neurons in LC was identified by standard criteria [51]: (1) long-lasting action potentials (> 2 ms); (2) spontaneous firing at a regular rhythm; (3) characteristic spikes with a long-lasting positive–negative waveform; (4) phasic activation by pressure applied to the hind paw followed by a longer period of post-activation inhibition; (5) a slow firing rate of 0.5–5 Hz and (6) the inhibition by the adrenergic-α2 receptor agonist medetomidine (1 mg/kg, i.p.) and subsequent reversal by the adrenergic-α2 receptor antagonist idazoxan (1 mg/kg, i.p.) [52]. An additional clue to the correct positioning of the electrode within the LC was the electrical silence immediately dorsal to LC, due to the IVth cerebral ventricle [53].

Once an action potential was isolated, spontaneous activity was first assessed for 5 min, then the characterization of NA neurons in LC was determined by passing the electrode five to eight times through four different areas within the LC, according to the method described by Blier and de Montigny [54]. The extracellular neuronal signal was filtered (bandpass 500–5000 Hz) and amplified with a computer connected to a CED Micro 1401 interface. Active neurons recorded from each mouse were analyzed according to the above defined criteria using the computer software Spike2 (script w_burst.s2s) (Cambridge Electronic Design, Cambridge, UK) [55]. The tonic activity of LC neurons was measured in an interval of 200 s as the mean ratio of the number of spikes within a burst (a train of at least two spikes with the first inter-spike interval of 20 ms and a final interval of 160 ms, [56] and the sum of recorded spikes (%). The spontaneous activity of LC neurons in 200 s and the total number of neurons found in the LC of sham and SNI mice was also considered.

### Immunofluorescence

Mice were deeply anesthetized and transcardially perfused with a saline solution followed by 4%paraformaldehyde in 0.1-M phosphate buffer [57]. Staining was performed by incubating slides with primary antibody (rabbit anti-TH, 1:500, abcam, UK) over night, followed by incubation with secondary antibody (Alexa fluor 488, Invitrogen 1:1000). The total number of TH-expressing neurons in each LC was estimated taking into account the cell group contained in 7 * 10 μm^2^. For each animal, the number of TH-positive neurons were counted from the right and left LC and the mean between both sides was done for each mouse. Pictures were performed using Leica microscope.

### Drugs

Forskolin (Cat. F6886), thapsigargin (Cat. T9033), medetomidine, idazoxan, and kolliphor were purchased from Sigma Aldrich (Milan, Italy). Histamine dihydrochloride (Cat. 3545), Immepip (Cat. 0932) were purchased from Tocris (UK). 2-Pentadecyl-2-oxazoline (PEA-OXA) was kindly provided by EPITECH Group SpA, Saccolongo (PD). PEA-OXA was dissolved in kolliphor 5% in saline. PEA-OXA (10 mg/kg) or vehicle (kolliphor 5%) were intraperitoneally daily administered starting 14 days after SNI or sham surgery for 16 days (day 30 from the SNI or sham surgery). The dosage of PEA-OXA was chosen based on previous studies of our and other laboratories [30, 58].

### Statistical analysis

Data were represented as mean ± SEM. The Kolmogorov–Smirnov test was used to examine the distribution of data from all experiments. In vitro analysis was performed by using Anova, followed by Tukey or Bonferroni test. All behavioral data were analyzed by using two-way ANOVA. P values < 0.05 were considered statistically significant. Statistical analysis was performed using Prism/Graphpad 6.0software (GraphPad Software, Inc.).

## Results

### *Evaluation of PEA-OXA activity on histamine receptors: *in vitro* and *in vivo* approaches*

Due to the role of histamine in neuropathic pain, we decided to explore the potential effect of PEA-OXA on the histamine receptors H1, H3 and H4. We generated a heterologous in vitro model by stably transfecting fibroblast-like COS-7 cells with a plasmid encoding for the human histamine H3 receptor. Since H3 and H4 receptors are coupled to Gαi/o proteins, and their stimulation leads to the inhibition of adenylyl cyclase (AC), with consequent reduction of cyclic adenosine monophosphate (cAMP) levels [59], we evaluated in both control (scramble) and histamine H3 or H4 receptor-overexpressing cells whether the treatment with increasing concentrations of PEA-OXA could change the intracellular levels of cAMP triggered by forskolin 10 µM. To select the concentration of PEA-OXA to be used, preliminary experiments were conducted measuring the viability of H3 or H4 receptor-transfected COS-7 cells in the presence of increasing concentrations of PEA-OXA (up to 10 µM). As shown in Additional file 1: Figure S1, only at 10 µM we observed a slight decrease in cell viability. However, to avoid any misinterpretation of results, PEA-OXA and, as a positive control, Histamine were used up to 3 µM and 30 µM, respectively. Our results show that in H3 transfected COS-7 cells, PEA-OXA (0.1–3 μM) dose-dependently partially inhibited the forskolin-induced stimulation of cAMP (Fig. 1a). Remarkably, in histamine H4 receptor-transfected cells, PEA-OXA was ineffective, also at 3 µM (Additional file 2: Fig. S2). Subsequently, the effect of PEA-OXA was evaluated in the histamine H1 receptor-transfected COS cells. In this case, being histamine H1 receptor coupled to Gq protein, we measured the effect of PEA-OXA on intracellular Ca^2+^ determined by using Fluo-4. As shown in Additional file 2: Figure S2, the stimulation of histamine H1 receptors with histamine or thapsigargin led to a robust increase in [Ca^2+^]i that was not prevented by the pre-incubation with PEA-OXA up to 5 µM. In summary, our results demonstrate that PEA-OXA shows activity only in histamine H3, and not H4 or H1 receptor-transfected cells, by preventing the forskolin-induced cAMP production, and thus acting as an agonist for this class of receptors.

In order to corroborate the in vitro results and to better understand the dynamic nature of the PEA-OXA (agonist, partial agonist) we performed the open field task in freely moving naïve mice by microinjecting into the third ventricle (I3V) the selective H3-agonist Immepip alone or in combination with PEA-OXA (Fig. 1b).

Naϊve mice microinjected with Immepip 3 nmol/0.3 µl in I3V showed a significant decrease in the travelled distance (1708 ± 371.2 cm, one-way ANOVA followed by Holm-Sidak post-hoc test) compared with naϊve mice treated with aCSF [4343 ± 354.4 cm, p < 0.0001, F_(3,16)_ = 18.21]. By contrast, naϊve mice that received PEA-OXA 2.5 nmol/0.3 µl microinjection in I3V did not show an increase in the ambulatory activity 5077 ± 352.7 cm, two-way ANOVA followed by Holm-Sidak post-hoc test) compared with naϊve mice that received aCSF. The co-injection of Immepip and PEA-OXA, at the dose of 3 and 2.5 nmol/0.3 μl, respectively, counteracted the hypokinetic effect induced by immepip alone (3888 ± 271.0 cm, two-way ANOVA followed by Holm-Sidak post-hoc test) compared with naϊve mice that received immepip alone [1708 ± 371.2 cm, one-way ANOVA followed by Holm-Sidak post-hoc test], confirming the H3-mediated effect of PEAOXA (Fig. 1c). This pharmacological effect of PEA-OXA is likely to show a partial agonist activity, since it behaves as an antagonist in presence of a pure, potent agonist. Due to the complex dynamic of this drug and, considering the mainly presynaptic localization of the H3 receptor into the brain, we performed in vivo microdialysis in sham and neuropathic mice with or without PEA-OXA treatment.

Intriguingly, we found that, in sham conditions, PEA-OXA increased the histamine release, whereas in the SNI mice, that already showed an enhancement of the histamine, reduced it. Briefly, the extracellular levels of histamine were significantly increased in SNI mice (635.7 ± 71.48 fmol/10 μl) chronically treated with vehicle compared to sham mice (233.3 ± 22.25 fmol/10 μl) receiving the same treatment (Fig. 1d).

The chronic treatment with PEA-OXA (10 mg/kg, i.p.) significantly increased the levels of histamine (HA) in sham mice (258.4 ± 28.84 fmol/10 μl), whereas it decreased them in SNI mice (828.9 ± 168.1 fmol/10 μl, Two-way ANOVA followed by Tukey’s post-hoc test Interaction: p < 0.0001, F_(1,28)_ = 44.11; Model p = 0.88 F_(1,28)_ = 0.02; Treatment p = 0.58 F_(1,28)_ = 0.30) compared to SNI mice treated with vehicle (Fig. 1d). These biochemical data, further suggest a complex dynamic profile of the PEA-OXA, possibly acting as a partial agonist.

### *Theoretical complexes of the human histamine H*_*3*_* receptor with histamine and PEA-OXA*

**Fig. 2:**
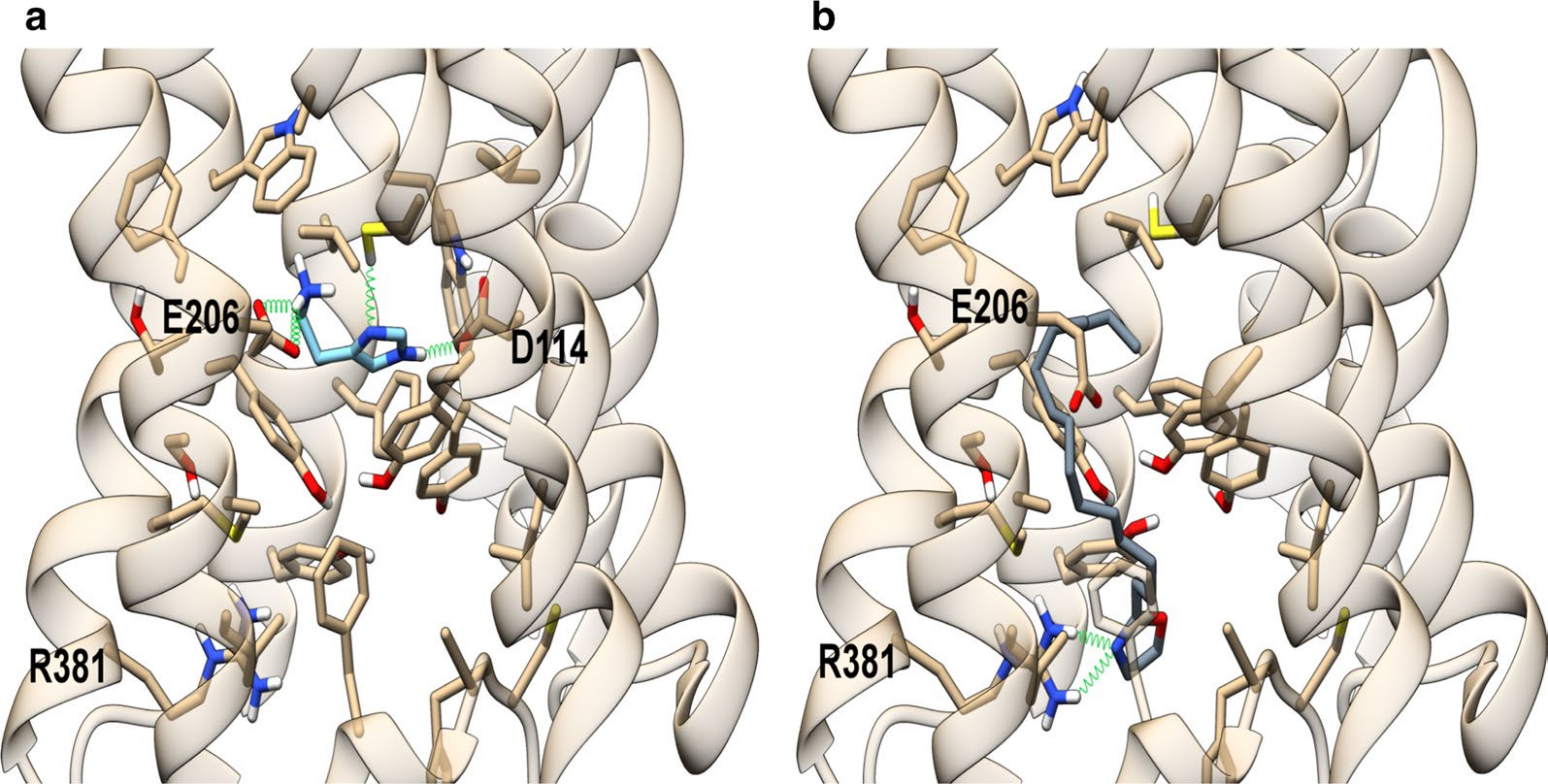
Structural alignment used for model building. Ballesteros–Weinstein numbers are used to label the most conserved residues within transmembrane helices, highlighted in cyan. Conserved cysteine residues involved in disulfide bridge are colored in yellow. The negatively charged residues Asp144 and Glu206 critical for histamine binding are colored in red while Arg381, proposed as relevant for PEA-OXA binding is colored in green. The long loop i3 was truncated in the model, leaving only the residues allowing a proper linker between the helices. The transmembrane helices H1–H8 are underlined while the intracellular and extracellular loops are labeled “i1–3” and “e1–3”, respectively

To rationalize the agonist activity of PEA-OXA at the H3 receptor (H_3_R), a theoretical model of this receptor was built as described in detail in the “Materials and methods” section, using the x-ray structure of the muscarinic M_3_ receptor (PDB id: 4U15), identified as best template (sequence identity of ~ 29%) by a sequence search using PSI-BLAST against PDB database. The structural alignment used for model building is shown in Fig. 2. Histamine H3 receptor shares with muscarinic M_3_ receptor two disulfide bridges: one, well conserved among class A GPCRs, connecting the extracellular loop 2 (e2) to helix 3, the other, peculiar of these receptors, involving two cysteine residues within loop e3, which links helix 6 to helix 7. This additional disulfide bridge also occurs in histamine H1, but not in H4, receptors.

**Fig. 3:**
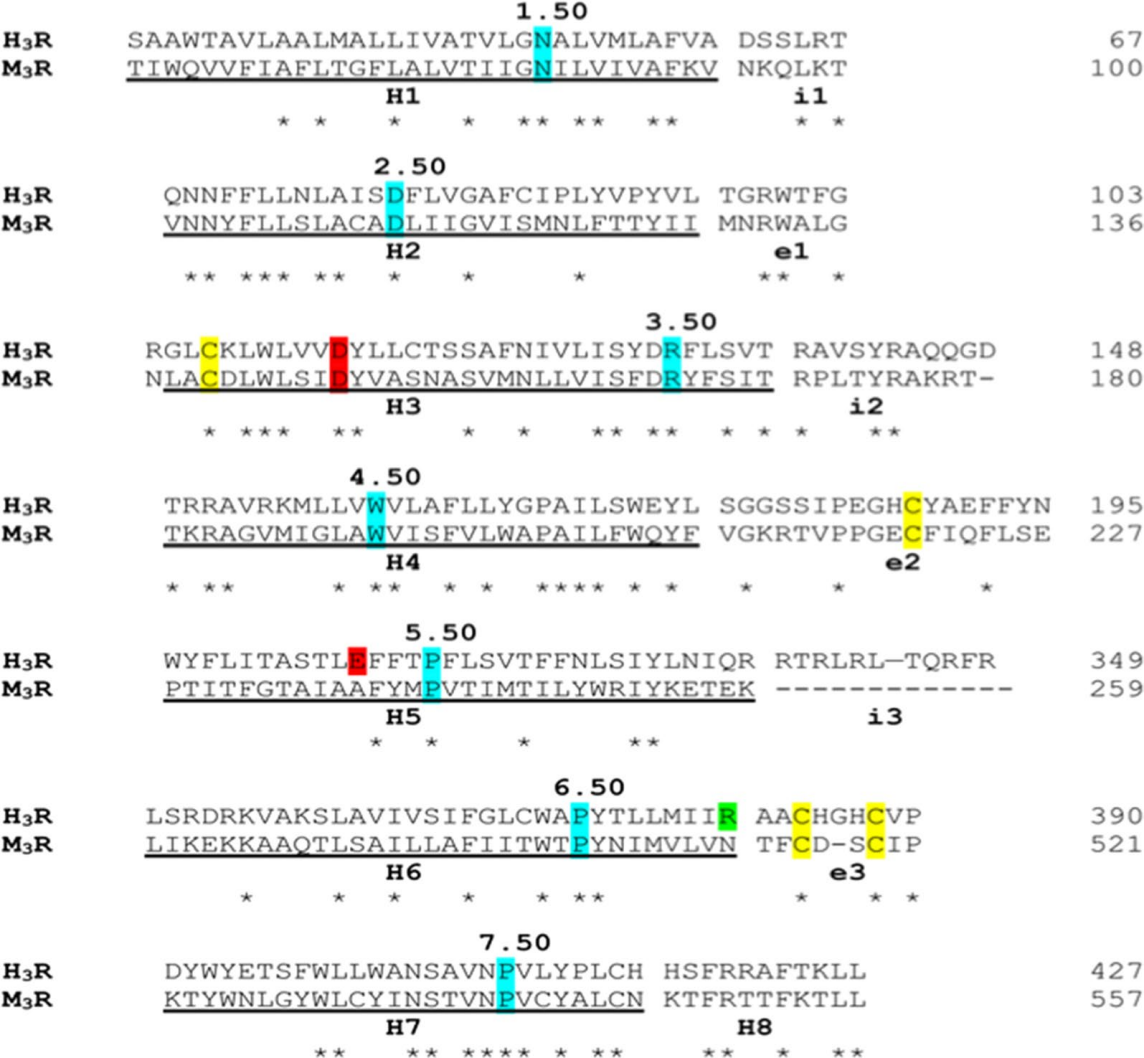
Energy minimized theoretical complexes of histamine H3 receptor with histamine (**a**) and PEA-OXA (**b**). Protein is colored in tan, histamine in light blue and PEA-OXA in slate gray. Ligands and residues within 5 Å from the ligands are shown in stick representation. Oxygen, sulfur and nitrogen heteroatoms are colored in red, yellow and blue, respectively. H-bonds are represented as green springs

To validate both the target model and the docking protocol, the endogenous ligand histamine was docked in the receptor binding site (Fig. 2). The best binding pose in terms of binding energy is shown in Fig. 3. It well recapitulates the binding mode already observed in other theoretical complexes [60] and validated by mutagenesis [59]. In this model, the positively-charged group of histamine forms ionic interactions with Glu206^5.46^ on helix 5, while the imidazole ring engages H-bonds with Asp114^3.32^ and a π-stacking with Tyr115^3.33^ on helix3. Instead, the best-scoring binding pose of PEA-OXA, well convergent among the different docking runs as described in detail in ““Materials and methods”” section, adopts a completely different orientation, since the oxazoline ring is involved in H-bonds with Arg381^6.58^ on helix 6 and in hydrophobic interactions with Tyr394^7.35^ on helix 7, while the alkyl chain extends toward a hydrophobic region formed by residues lying on helices 3, 5, 6 and the loop e2, that is Leu111^3.29^, Tyr115^3.33^, Cys118^3.36^, Ala122^3.40^, Phe192 (loop e2), Phe197^5.38^, Leu198^5.39^, Ala202^5.42^, Ser203^5.43^, Phe207^5.47^, Trp371^6.48^, Tyr374^6.51^, Thr375^6.52^, Met378^6.55^. Our results are in agreement with the well-known key role of helix 6 in the activation of GPCRs class A and with previous studies that highlighted the critical role of such hydrophobic pocket, adjacent to the histamine binding site, and, in particular, the aforementioned residues on helix 6, in histamine H3 receptor activation induced by agonists featuring a lipophilic tail in place of the terminal positively charged nitrogen atom [60]. Moreover, the role of Arg381^6.58^ in histamine H3 receptor binding has been already reported for a series of piperazine derivatives, which showed an increased binding affinity when a carbonyl group occurred in the lipophilic part of the molecules [61]. It is noteworthy that Arg381^6.58^ corresponds to a residue of isoleucine and leucine in histamine H1 and H4 receptor, respectively, and this lack of conservation could explain the observed selectivity of PEA-OXA for H3 over H1 and H4 receptors (Fig. 3). To the best of our knowledge, PEA-OXA represents the first example of a lipophilic histamine H3 receptor agonist devoid of any proton donor and/or positively charged group.

### Effect of chronic treatment with vehicle or PEA-OXA on tactile allodynia

**Fig. 4:**
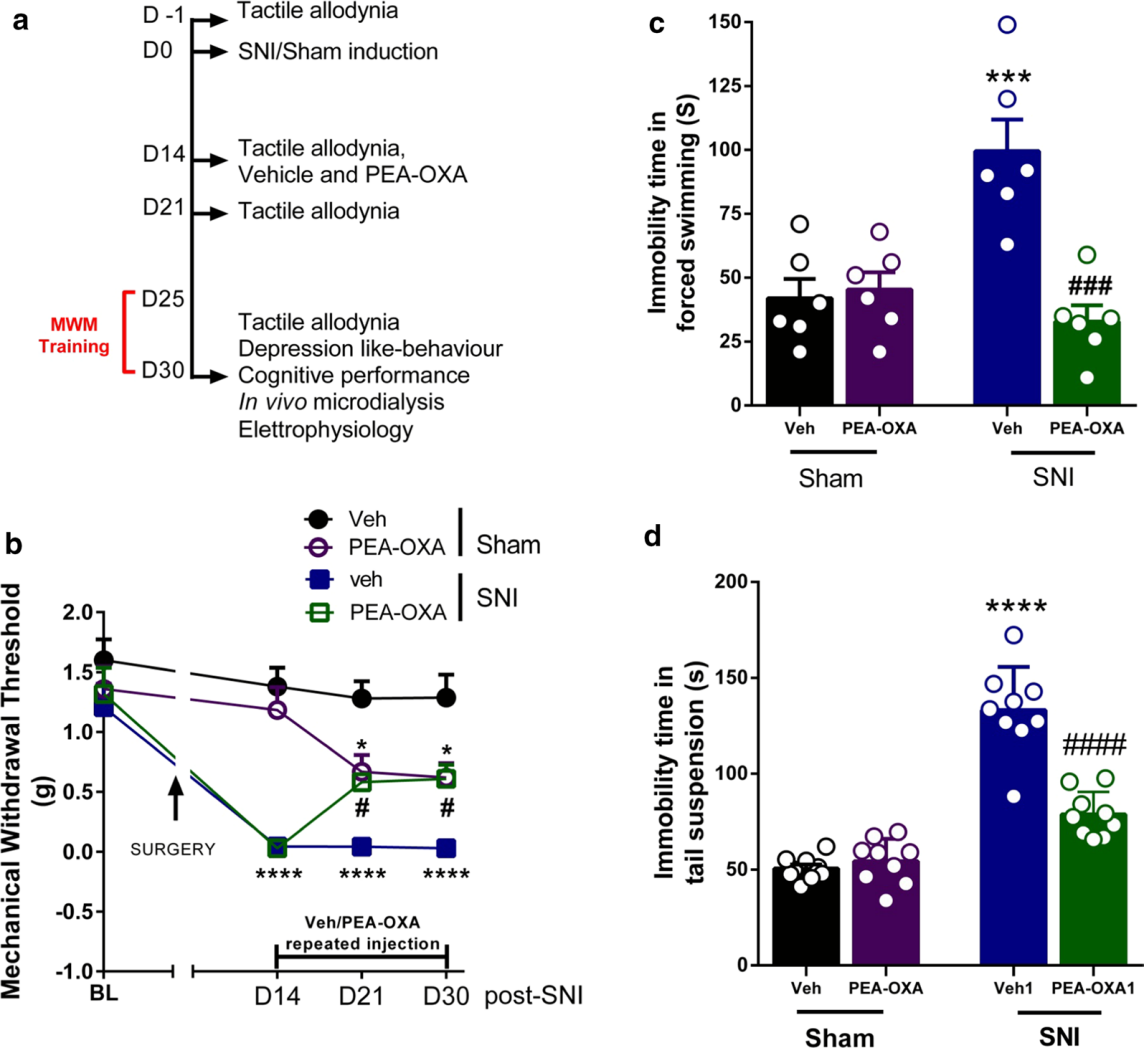
Effect of the chronic treatment with vehicle (kolliphor 5% in saline, v/v, i.p.) or PEA-OXA (10 mg/kg, i.p.) on pain and depression-like behavior in sham and SNI mice. **a** The timeline of the sham or SNI surgery, behavioral, microdialysis, electrophysiology experiments and vehicle or PEA-OXA chronic treatment. **b** The effect of the chronic treatment with vehicle or PEA-OXA on mechanical allodynia. **c**, **d** The effect of vehicle or PEA-OXA on duration of immobility in the forced swimming or tail suspension tests, respectively. Experiments have been carried out 30 days after SNI or sham surgery. Each point represents the mean ± S.E.M. Two-way ANOVA or RM Two-way ANOVA, followed by Tukey's post hoc test for multiple comparisons test was used for statistical analysis. p < 0.05 was considered statistically significant. Symbols indicate significant differences: *vs Sham/veh (p < 0.05), ***vs Sham/veh (p < 0.001), ****vs Sham/veh (p < 0.0001), ^#^vs SNI/veh (p < 0.05), ^###^vs SNI/veh (p < 0.001), ^####^vs SNI/veh (p < 0.0001), respectively

A diagram summarizing the time course of sham or SNI surgery, treatment and timing of allodynia measurements is shown in Fig. 4a. SNI mice showed a significant reduction of the MWT of the ipsilateral hind paw compared to the sham mice 14 days after the surgery (Sham: 1.38 ± 0.16 g, SNI: 0.04 ± 0.01 g, followed by two-way ANOVA Tukey’s post-hoc test). The chronic treatment with vehicle from 7 to 16 days did not change the MWT in both sham and SNI mice (21 days after surgery, Sham: 1.28 ± 0.14 g, SNI: 0.044 ± 0.01 g; 30 days after surgery, Sham: 1.28 ± 0.19 g, SNI: 0.03 ± 0.01 g; followed by two-way ANOVA Tukey’s post-hoc test The chronic treatment with PEA-OXA (10 mg/kg, i.p.) from 7 to 16 days changed the MWT in the ipsilateral hind paw in both sham and SNI mice (21 days after surgery, Sham: 0.67 ± 0.14 g, SNI: 0.58 ± 0.06 g; 30 days after surgery, Sham: 0.6 ± 0.12 g, SNI: 0.61 ± 0.12 g; two-way ANOVA followed by Tukey’s post-hoc test p < 0.0001, F_(3,36)_ = 50.67). In sham mice, chronic treatment with PEA-OXA (10 mg/kg, i.p.) for 7 and 16 days significantly decreased the MWT, while in SNI mice the same treatment increased the MWT compared to sham and SNI mice treated with vehicle, respectively (Fig. 4b). No change was observed in the PWT of the contralateral paw in SNI or sham mice (data not shown).

### Effect of chronic treatment with vehicle or PEA-OXA on depression-like behavior

In the forced swimming test, SNI mice treated with vehicle showed a significant increase in the duration of immobility compared to sham mice receiving the same treatment (Sham: 42.00 ± 7.49 s, SNI: 99.50 ± 12.41 s, Two-way ANOVA followed by Tukey’s post-hoc test Interaction: p < 0.0006 F_(1,20)_ = 16.50; Model p < 0.0167 F_(1,20)_ = 6.817; Treatment p < 0.015 F_(1,20)_ = 13.50). The chronic treatment with PEA-OXA (10 mg/kg, i.p) for 16 days did not change the duration of immobility in sham mice (45.33 ± 6.81 s) whereas reduced it significantly in SNI mice (32.83 ± 6.36 s) compared to SNI group treated with vehicle (Fig. 4c). In the tail suspension test, SNI mice treated with vehicle showed a significant increase in the duration of immobility compared to the sham mice receiving the same treatment (Sham: 50.57 ± 2.05 s, SNI: 133.2 ± 7.49 s, Two-way ANOVA followed by Tukey’s post-hoc test Interaction: p < 0.0001 F_(1,32)_ = 37.22; Model p < 0.0001 F_(1,32)_ = 125.9; Treatment p < 0.0001 F_(1,32)_ = 28.16). The chronic treatment with PEA-OXA (10 mg/kg, i.p.) for 16 days did not change the duration of immobility in sham mice (54.36 ± 3.90 s) whereas it reduced it significantly in SNI mice (78.77 ± 3.93 s) compared SNI group treated with vehicle (Fig. 4d).

### Effect of chronic treatment with vehicle or PEA-OXA on anxiety-like behavior

In the elevated plus-maze test, SNI mice treated with vehicle showed a significant decrease in the open arm choice compared to sham mice receiving the same treatment (Sham: 56.37 ± 1.20%, SNI: 19.84 ± 4.17%, Two-way ANOVA followed by Tukey’s post-hoc test Interaction: p = 0.06 F_(1,20)_ = 3.822; Model p < 0.0001 F_(1,20)_ = 175.8; Treatment p = 0.29 F_(1,20)_ = 1.16) The treatment with PEA-OXA (10 mg/kg, i.p.) for 16 days did not change the open arm choice in either SNI or sham mice (Sham: 54.26 ± 1.72%, SNI: 26.37 ± 1.22%) (Additional file 3: Fig. S3A).

In the light dark box test, SNI mice that received vehicle administration showed a significant increase in the time spent in the dark box compared to sham mice receiving vehicle treatment. (Sham: 412.2 ± 27.89 s, SNI: 563.7 ± 9.67 s, Two-way ANOVA followed by Tukey’s post-hoc test Interaction: p = 0.01 F_(1,20)_ = 8.049; Model p = 0.02 F_(1,20)_ = 6.43; Treatment p = 0.10 F_(1,20)_ = 2.86). The chronic treatment with PEA-OXA (10 mg/kg, i.p.) for 16 days significantly increased the time spent in dark box in sham mice (444.5 ± 30.74 s) compared to sham mice treated with vehicle and decreased the time spent in dark box in SNI mice (436.0 ± 36.94 s) compared to SNI mice treated with vehicle (Additional file 3: Fig. S3B). In the marble burying test, SNI mice receiving a chronic treatment with vehicle showed a significant increase in the number of diggings and marbles buried compared to sham mice receiving the same treatment (Diggings, Sham: 218.7 ± 36.18, SNI: 469.2 ± 45.72, Two-way ANOVA followed by Tukey’s post-hoc test Interaction: p = 0.01 F_(1,20)_ = 8.03; Model p = 0.10 F_(1,20)_ = 2.88; Treatment p = 0.05 F_(1,20)_ = 4.52; Marbles, Sham: 2 ± 0.58, SNI: 8.50 ± 1.06, Two-way ANOVA followed by Tukey’s post-hoc test Interaction: p = 0.003 F_(1,20)_ = 11.54; Model p = 0.12 F_(1,20)_ = 2.57; Treatment p = 0.06 F_(1,20)_ = 3.95). The chronic treatment with PEA-OXA (10 mg/kg, i.p.) for 16 days increased the number of diggings and marble buried in sham mice (Diggings: 492.8 ± 58.90; marbles: 9 ± 1.69), while it did not change significantly the number of diggings and marbles in SNI mice (Diggings: 430.0 ± 73.17; marbles: 6.67 ± 1.56), compared to sham and SNI mice receiving vehicle treatment, respectively (Additional file 3: Fig. S3C and D).

### Effect of chronic treatment with vehicle or PEA-OXA on cognitive performance

**Fig. 5:**
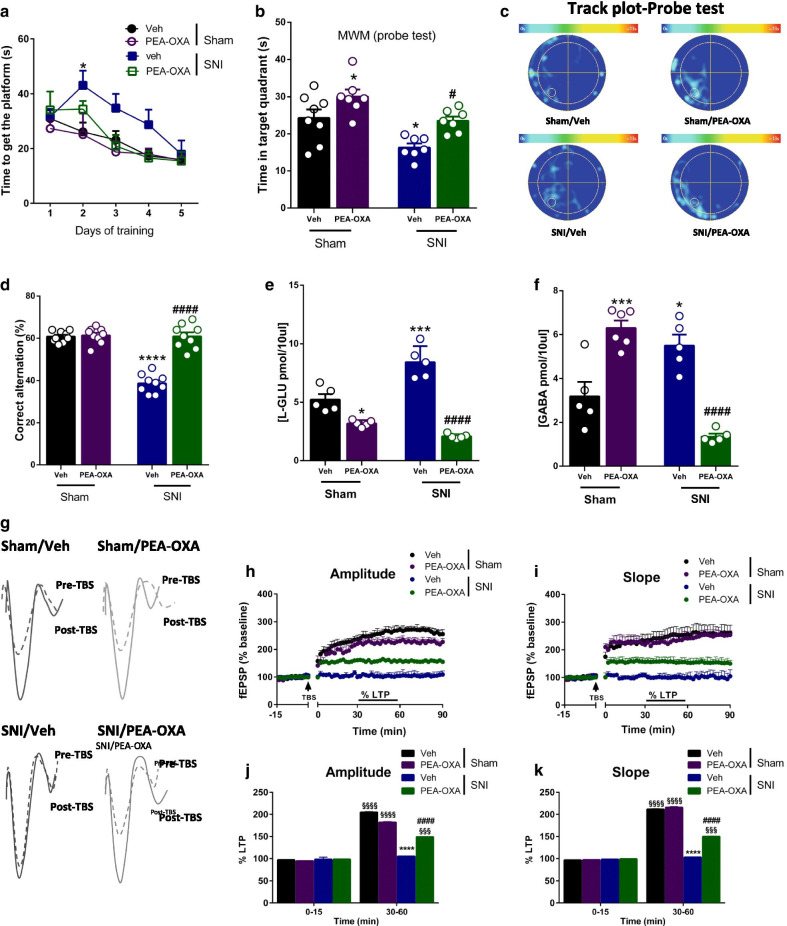
Effect of the chronic treatment with vehicle (kolliphor 5% in saline, v/v, i.p.) or PEA-OXA (10 mg/kg, i.p.) on cognitive behavior, the release of amino acids and DG extracellular recordings in sham and SNI mice. **a** The effect of vehicle or PEA-OXA on the latency to reach the platform in sham and SNI mice during the 5 days of training phase in the Morris water maze (MWM). **b** The effect of vehicle or PEA-OXA on the time spent in the target quadrant in the MWM probe test in sham and SNI mice. **c** The effect of vehicle or PEA-OXA on the track-plot occupancy in the MWM probe test in sham and SNI mice. **d** The effect of vehicle or PEA-OXA on the percentage of alternations in the y-maze. **e**, **f** The effect of vehicle or PEA-OXA on the extracellular level of glutamate (l-Glu) and GABA in the DG of sham and SNI mice. **g** Representative traces recorded before (dashed line) and after (full line) the induction of the LTP (time 0) in the DG of sham and SNI mice, treated with vehicle or with PEA-OXA. Data are represented as mean ± SEM. Two-way ANOVA, followed by Tukey's post hoc test for multiple comparisons test was used for statistical analysis. p < 0.05 was considered statistically significant. Symbols indicate significant differences: *vs Sham/veh (p < 0.05), ***vs Sham/veh (p < 0.001), ****vs Sham/veh (p < 0.0001), ^#^vs SNI/veh (p < 0.05), ^###^vs SNI/veh (p < 0.001), ^####^vs SNI/veh (p < 0.0001), respectively. **h**–**k** The fEPSPs amplitude and slope recorded before and after the induction of the LTP (time 0) in the DG of sham and SNI mice treated with vehicle or PEA-OXA. LTP magnitude was calculated as a percentage of the baseline between the last 40 and 60 min of recording. Data are represented as mean ± SEM. RM Two-way ANOVA, followed by Dunnett’s or Tukey's post hoc tests for multiple comparisons tests were performed for comparison vs PRE-TBS and among groups during the time course, respectively. Symbols indicate significant differences: ^§§§^vs PRE-TBS (0–15 min) (p < 0.001), ^§§§§^vs PRE-TBS (0–15 min) (p < 0.0001), ****vs Sham/veh (p < 0.0001), ^####^vs SNI/veh (p < 0.0001), respectively

In the Morris water maze test, which measures spatial memory, SNI mice treated with vehicle showed a significant increase in the latency to reach the platform during the second day of the training phase compared to sham mice receiving the same treatment (Sham: 26.12 ± 3.37 s, SNI: 43.08 ± 5.29 s, followed by two-way ANOVA Tukey’s post-hoc test Interaction: p = 0.3092 F_(12,96)=_ 1.179; Time: p < 0.0001 F_(4,96)_ = 14.03; Treatment: p = 0.0369 F_(3,24)_ = 3.317). The chronic treatment with PEA-OXA (10 mg/kg, i.p.) did not change the time to reach the platform in either sham or SNI mice (At 5th day, Sham/vehicle: 15.98 ± 2.47 s, Sham/PEA-OXA: 15.71 ± 1.84 s, SNI/vehicle: 17.92 ± 5.02 s, SNI/PEA-OXA: 15.45 ± 3.30 s) (Fig. 5a). In the probe test, SNI mice treated with vehicle showed a decrease in the time spent in the target quadrant compared to sham mice receiving the same treatment (Fig. 5b) (Sham: 24.28 ± 2.28 s, SNI: 16.31 ± 1.10 s, Two-way ANOVA followed by Tukey’s post-hoc test Interaction: p = 0.96 F_(1,24)_ = 0.003; Model p = 0.0005 F_(1,24)_ = 16.03; Treatment p = 0.0002 F_(1,24)_ = 18.47). The chronic treatment with PEA-OXA (10 mg/kg, i.p.) significantly increased the time spent in the target quadrant in sham mice compared to Sham group treated with vehicle (30.06 ± 1.90 s), and in the SNI mice compared to SNI group treated with vehicle (23.51 ± 1.14 s) (Fig. 5b). The track plots recorded on day 6 are shown in Fig. 5c.

In the Y-maze test, which measures spatial working memory, SNI mice chronically treated with vehicle showed a significant decrease in the number of alternations compared to sham mice receiving the same treatment (Fig. 5d) (Sham: 60.78 ± 0.86%, SNI: 38.67 ± 1.46%, Two-way ANOVA followed by Tukey’s post-hoc test Interaction: p < 0.0001 F_(1,32)_ = 59.46; Model p < 0.0001 F_(1,32)_ = 64.44; Treatment p < 0.0001 F_(1,32)_ = 65.72). The chronic treatment with PEA-OXA (10 mg/kg, i.p.) did not change the percentage of alternations in sham mice (61.33 ± 1.21%), whereas it increased in SNI mice (60.89 ± 1.88%) compared SNI group receiving vehicle (Fig. 5d).

### Effect of chronic treatment with vehicle or PEA-OXA on glutamate and GABA release

SNI mice chronically treated with vehicle showed a significant increase in glutamate (l-Glu) and GABA release compared to sham mice treated with vehicle (Fig. 5e, f) (l-Glu, Sham: 5.22 ± 0.47 pmol/10 µl, SNI: 8.43 ± 0.61 pmol/10 µl, Two-way ANOVA followed by Tukey’s post-hoc test Interaction: p < 0.0001 F_(1,16)_ = 29.65; Model p = 0.017 F_(1,16)_ = 7.07; Treatment p < 0.0001 F_(1,16)_ = 112.5; GABA, Sham: 3.19 ± 0.66 pmol/10 µl, SNI: 5.51 ± 0.49 pmol/10 µl, Two-way ANOVA followed by Tukey’s post-hoc test Interaction: p < 0.0001 F_(1,16)_ = 62.98; Model p = 0.009 F_(1,16)_ = 8.59; Treatment p = 0.32 F_(1,16)_ = 1.05). The chronic treatment with PEA-OXA (10 mg/kg, i.p.) decreased the extracellular level of l-Glu in sham and SNI mice (Sham: 3.19 ± 0.13 pmol/10 µl, SNI: 2.09 ± 0.10 pmol/10 µl) as compared to sham and SNI mice treated with vehicle (Fig. 5e). The chronic treatment with PEA-OXA (10 mg/kg, i.p.) also increased in sham mice and decreased in SNI mice (Sham: 6.30 ± 0.34 pmol/10 µl, SNI: 1.36 ± 0.13 pmol/10 µl) the extracellular levels of GABA compared to sham and SNI mice treated with vehicle (Fig. 5f).

### Effect of chronic treatment with vehicle or PEA-OXA on LTP in the LEC-DG pathway

Following theta burst stimulation (TBS) to the LEC sham mice chronically treated with vehicle showed LTP induction (amplitude: 205.02 ± 1.06%, slope: 211.49 ± 1.09%, 30–60 min post-TBS; n = 5) (Fig. 5g–i). LTP was not observed in SNI mice chronically treated with vehicle (amplitude: 105.23 ± 0.63%, slope: 103.27 ± 0.63%, 30–60 min post-TBS; n = 5) (Fig. 5g–i). Moreover, average I/O curves measured at 350 μA resulted in a significant shift of input–output curves towards a higher fEPSP amplitude (SNI: − 9.5 ± 0.7 mV, sham: − 4.5 ± 0.63 mV, n = 10) and slope (SNI: − 3.2 ± 0.45 mV/ms, sham: − 1.4 ± 0.33 mV, n = 10) in SNI mice as compared to sham mice both receiving vehicle treatment (Fig. 5g). No changes in LTP induction and maintenance were observed in sham mice chronically treated with PEA-OXA as compared to sham mice treated with vehicle (amplitude: 182.14 ± 1.51%, slope: 215.78 ± 1.67%, 30–60 min post-TBS; n = 5) (Fig. 5h–k). Chronic treatment of SNI mice with PEA-OXA (10 mg/kg, i.p.) instead restored LTP, which was maintained for at least 90 min post-TBS. In particular, fEPSPs amplitude (148.97 ± 0.17%, 30–60 min post-TBS; n = 5) (F_1,38_ = 269; p < 0.0001 for factor time; F_3,38_ = 328; p < 0.0001 for factor treatment; F_3,38_ = 352; p < 0.0001 for time X treatment interaction) and slope (150.08 ± 0.92%, 30–60 min post-TBS; n = 5) (F_1,38_ = 12.931; p < 0.0001 for factor time; F_3,38_ = 1739; p < 0.0001 for factor treatment; F_3,38_ = 1851; p < 0.0001 for time X treatment interaction) significantly increased compared to SNI mice chronically treated with vehicle (Fig. 5h–k).

### Effect of chronic treatment with vehicle or PEA-OXA on norepinephrine and dopamine

**Fig. 6:**
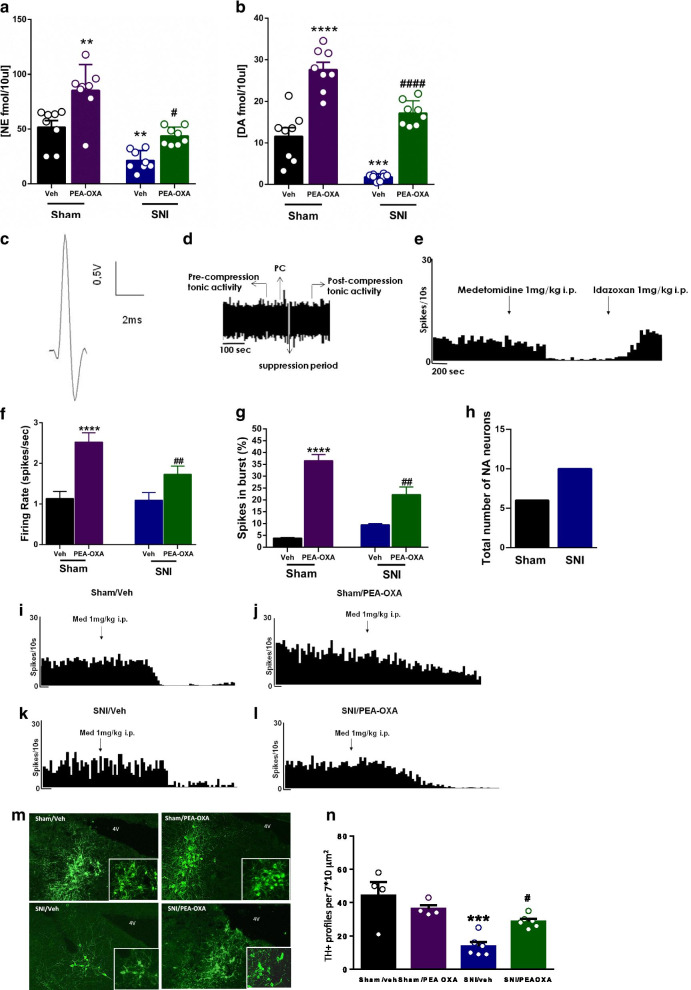
Effect of the chronic treatment with vehicle (kolliphor 5% in saline, v/v, i.p.) or PEA-OXA (10 mg/kg, i.p.) on the release of biogenic amines in the hippocampus CA3 and LC neuron activity in sham and SNI mice. **a**, **b** The effect of vehicle or PEA-OXA on the extracellular level of norepinephrine (NE) and dopamine (DA) in the CA3 of sham and SNI mice. **c** The characteristic spike of a noradrenergic neuron of the LC with a long-lasting positive–negative waveform. **d** Displays a phasic activation followed by a long period of post-activation inhibition induced by a nociceptive pressure applied to the hind paw (PC). **e** The effect of medetomidine, an adrenergic alpha2 receptor agonist, and idazoxan, an adrenergic alpha2 receptor antagonist on the firing rate in the LC. **f**, **g** show the effect of vehicle or PEA-OXA on the tonic activity of LC neurons, in terms of firing rate and percentage of spikes in burst, in sham and SNI mice. **h** The number of noradrenergic neurons found in sham and SNI mice. **i**–**l** Examples of ratemater records which illustrate the effect of medetomidine on the firing rate of LC neurons in sham and SNI treated with vehicle or PEA-OXA. **m**, **n** The TH positive profiles in the LC neurons and relative quantification. Data are represented as mean ± SEM. Two-way ANOVA, followed by Tukey's post hoc test for multiple comparisons test was used for statistical analysis. p < 0.05 was considered statistically significant. Symbols indicate significant differences: **vs Sham/veh (p < 0.01), ***vs Sham/veh (p < 0.001), ****vs Sham/veh (p < 0.0001) and ^#^vs SNI/veh (p < 0.05), ^##^vs SNI/veh (p < 0.01), ^####^vs SNI/veh (p < 0.0001), respectively

The extracellular levels of norepinephrine (NE), and dopamine (DA) were significantly reduced in SNI mice (NE: 21.31 ± 3.1 fmol/10 μl; DA: 1.77 ± 0.28 fmol/10 μl) chronically treated with vehicle compared to sham mice (NE: 51.74 ± 6.12 fmol/10 μl; DA: 11.57 ± 2.09 fmol/10 μl) receiving the same treatment (Fig. 6a, b). The chronic treatment with PEA-OXA (10 mg/kg, i.p.) significantly increased the levels of NE and DA in both sham and SNI mice compared to sham and SNI mice treated with vehicle as shown in Fig. 6a, b (NE, Sham: 85.34 ± 8.29 fmol/10 μl; SNI: 43.75 ± 2.88 fmol/10 μl Two-way ANOVA followed by Tukey’s post-hoc test Interaction: p = 0.32 F_(1,28)_ = 0.99; Model p < 0.0001 F_(1,28)_ = 41.57; Treatment p < 0.0001 F_(1,28)_ = 25.17; DA, Sham: 27.58 ± 1.79 fmol/10 μl; SNI: 17.16 ± 1.05 fmol/10 μl Two-way ANOVA followed by Tukey’s post-hoc test Interaction: p = 0.83 F_(1,28)_ = 0.046; Model p < 0.0001 F_(1,28)_ = 46.50; Treatment p < 0.0001 F_(1,28)_ = 112.1)

.

### Effect of chronic treatment with vehicle or PEA-OXA on LC neuron activity

**Fig. 7:**
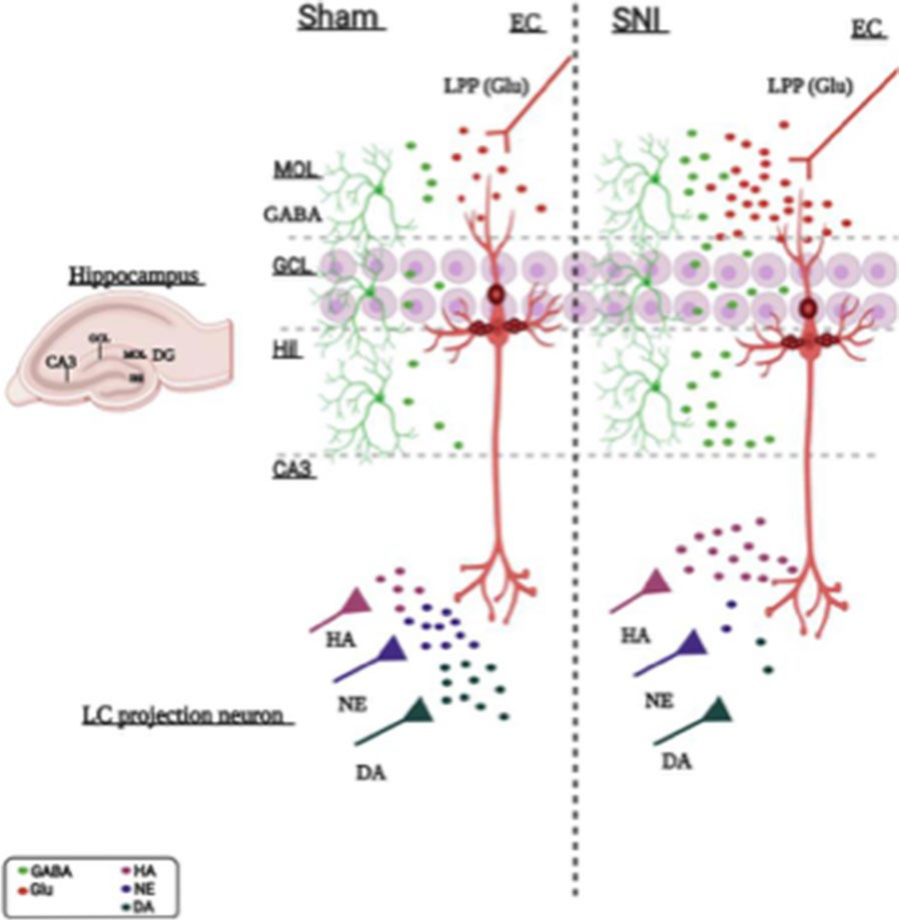
The illustration shows the different anatomical and neurochemical condition in the different subregions of the hippocampal DG and CA3 in sham and SNI mice. In the DG of SNI mice there is an increase in glutamate (Glu) and a reduction in GABA levels in the molecular layer (MOL), granular cell layer (GCL), and hilus (Hil) of the DG. In the SNI mice an increase in levels of histamine and a reduction in those of dopamine and norepinephrine are observed in the CA3, where the projections of catecholaminergic and histaminergic neurons arrive from the LC. The glutamatergic pyramidal neuron, the glutamatergic projection fiber from the entorhinal cortex (EC, lateral perforating path, LPP) and the extracellular glutamate are painted in red, the GABAergic interneurons and the extracellular GABA in green, the histaminergic (HA), noradrenergic (NE) and dopaminergic (DA) projection neurons are painted in pink, purple and dark green, respectively. A representation of the organization of the different hippocampal subregions is shown in the left panel

**Fig. 8:**
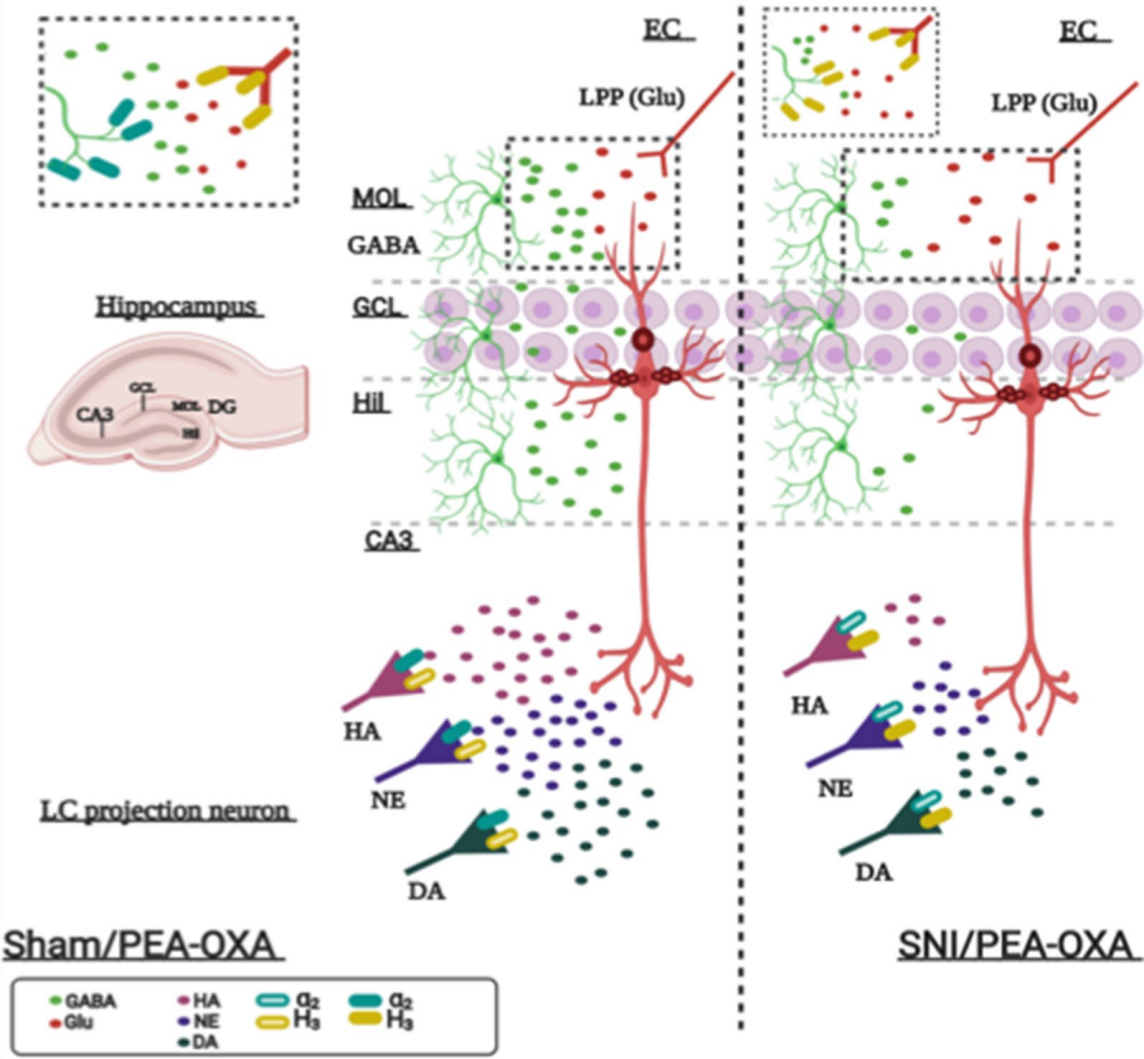
The illustration shows the effect of PEA-OXA on the release of amino acids and biogenic amines in the hippocampus DG and CA3 in sham and SNI mice. In sham mice (left), chronic treatment with PEA-OXA reduces glutamate and increases GABA release in the DG. These effects are due to the action of PEA-OXA as a partial agonist on histamine H3 heteroreceptors, yellow filled rectangles and as an antagonist on alpha2 adrenergic heteroreceptors, blue filled rectangles, respectively on the glutamatergic and GABAergic terminals. An enlargement corresponding to the dotted box is shown at the top left. In the CA3 of sham mice PEA-OXA causes an increase in the levels of histamine, dopamine and norepinephrine, an action due to the blocking of the adrenergic alpha2 heteroreceptor. In SNI mice (right), PEA-OXA reduces the levels of glutamate (Glu) and GABA in the molecular layer (MOL), granular cell layer (GCL) and hilus (Hil) of the DG, an effect due to the stimulation of the histamine H3 heteroreceptor. An enlargement corresponding to the dotted box is shown at the top left. In the CA3 of SNI mice the chronic treatment with PEA-OXA decreases (and normalizes) the levels of histamine while increases (and normalizes) those of dopamine and norepinephrine, an action due to the stimulation of histamine H3 autoreceptor and heteroreceptor on histaminergic and cathecolaminergic terminals, respectively, arriving from LC. The PEA-OXA, being a partial agonist in presence of high levels of histamine observed in the SNI, acts as an antagonist. The glutamatergic pyramidal neuron, the glutamatergic projection fiber from the entorhinal cortex (lateral perforating path, LPP) and the extracellular glutamate are painted in red, the GABAergic interneurons and the extracellular GABA in green, the histaminergic (HA), noradrenergic (NE) and dopaminergic (DA) projection neurons are painted in pink, purple and dark green, respectively. A representation of the organization of the different hippocampal subregions is shown in the left panel

LC neurons of sham mice chronically treated with vehicle showed a characteristic waveform (Fig. 6c) and patterns of activity as previously described in healthy rats [51] and mice [52]. The tonic activity of LC neurons was evaluated in terms of firing rate (spikes/s) and percentage of spikes in burst (%), which are the main electrophysiological parameters involved in the release of noradrenaline. Moreover, to identify adrenergic α2 receptor function, medetomidine (1 mg/kg, i.p) and idazoxan were also administrated (Fig. 6e). Sham mice chronically treated with vehicle showed an ongoing activity of 1.13 ± 0.17 spikes/s (Fig. 6f), and a percentage of spikes in burst of 3.89 ± 0.2% (Fig. 6g) of LC neurons. SNI mice chronically treated with vehicle showed no differences in the spontaneous firing rate nor in burst firing percentage (1.1 ± 0.19 spikes/s and 9.4 ± 0.53%, respectively) compared to the sham mice receiving the same treatment (Fig. 6f, g). Chronic administration with PEA-OXA (10 mg/kg i.p.) induced a significant increase in tonic activity of LC neurons in both sham and SNI mice compared to sham and SNI mice chronically treated with vehicle (Fig. 6f, g). Sham mice chronically treated with PEA-OXA (10 mg/kg, i.p.) displayed a firing rate of 2.52 ± 0.23 spikes/s and the percentage of spikes in burst of 36.54 ± 2.54%. SNI mice chronically treated with PEA-OXA (10 mg/kg, i.p.) showed a firing rate of 1.73 ± 0.20 spikes/s and percentage of spikes in burst of 22.24 ± 3.2% as shown in Fig. 6f, g (Two-way ANOVA followed by Tukey’s post-hoc test; Firing rate: Interaction p = 0.07 F_(1,16)_ = 3.52; Model p = 0.05 F_(1,16)_ = 4.36; Treatment p < 0.0001 F_(1,16)_ = 25.68; Spike in burst: Interaction p = 0.0002 F_(1,16)_ = 22.98; Model p = 0.05 F_(1,16)_ = 4.47; Treatment p < 0.0001 F_(1,16)_ = 120.70). Finally, we found a substantial difference in the number of LC neurons in sham and SNI mice chronically treated with vehicle. Indeed, SNI mice showed an increased number of LC neurons as compared to sham (Fig. 6h–l) suggesting a possible different response of adrenergic α2-receptor stimulation by PEA-OXA in the two groups of mice. Finally, we found that the TH positive profiles were significantly reduced in the SNI as compared to the sham mice. The repeated administration of PEA-OXA restore the TH-expressing neurons in the Locus Coeruleus (Fig. 6m, n). The illustration of the different anatomical and neurochemical condition in the different subregions of the hippocampal DG and CA3 in sham and SNI mice is shown in the scheme (Fig. 7) and the effect of PEA-OXA on the release of amino acids and biogenic amines in the hippocampus DG and CA3 in sham and SNI mice is shown in Fig. 8.

## Discussion

This study was undertaken to evaluate the effect of PEA-OXA on sensory, affective and cognitive alterations associated with the SNI model of neuropathic pain and to shed light on the possible mechanisms underlying these effects. PEA-OXA is a natural compound, which was first proposed to act via elevation of the tissue levels of *N*-palmitoylethanolamine (PEA) [31]. Indeed, PEA-OXA was found to inhibit the PEA catabolic enzyme, *N*-acylethanolamine-hydrolyzing acid amidase (NAAA) [58, 62]. We recently showed that PEA-OXA binds the adrenergic alpha2 receptor by acting as an antagonist and can be a promising drug in the treatment of behavioral and cognitive impairments in a murine model of post-traumatic stress disorder, the mTBI [30]. In this study, we show that PEA-OXA, besides being an adrenergic alpha2 receptor antagonist, also exhibits partial agonist activity at histamine H3 receptors. As a multi-target drug, PEA-OXA could broaden its spectrum of action towards all the components of neuropathic pain and could represent a lead compound for the development of novel drugs effective towards neuropsychiatric diseases.

### PEA-OXA pharmacological modulation of histamine H3 receptor: in vitro and in vivo evidence

Our in vitro study has shown here that PEA-OXA, at the highest concentration used (3 μM): (i) prevented the forskolin dependent stimulation of cAMP in histamine H3 receptor transfected cells though not in histamine H4 receptor-transfected cells (ii) was not able to modify histamine- or thapsigargin-induced Ca^2+^ increase in cells transfected with histamine H1 receptors. As emerged from docking studies, PEA-OXA represents an “atypical” ligand of H3R since it adopts a completely different orientation from histamine, engaging H-bonds with Arg381 on helix 6, rather than interacting with the negatively charged residues Glu206 and Asp114. This result is not surprising since PEA-OXA is not positively charged and lacks any H-bond donor group. However, its binding mode is consistent with previous investigations which highlighted a critical role of a hydrophobic pocket adjacent to the histamine binding site in agonist-induced histamine H3 receptor activation [60]. We cannot exclude that PEA-OXA might behave as a positive allosteric modulator of the H3 receptors as also suggested by the docking data.

The best-known function of the histamine H3 receptor is the presynaptic regulation of the release of neurotransmitters such as histamine itself, noradrenaline, dopamine, acetylcholine, GABA, glutamate, serotonin, and some neuropeptides in the CNS [59]. Pharmacological studies initially performed to understand the role of histamine H3 receptor in pain processing, instead of clarifying this issue, brought more questions than answers. This perhaps was due to the pain model used, the nociceptive stimulus selected, together with different affinity and selectivity for the histamine receptors, and dose and routes of administration, of the molecule used [63–66]. However, in the last decade, more selective agents have shown that neuropathic pain is better and more persistently controlled by novel antagonists/inverse agonists at the histamine H3 receptor [24–26]. Furthermore, the pain killer effect of histamine H3 receptor antagonists/inverse agonists in peripheral neuropathy seems to be the consequence of the desensitization of the adrenergic alpha2 receptor in LC [25]. It is important to note, however, that PEA-OXA behaves as a partial agonist of H3 receptors, and yet it has shown a fair anti-allodynic action, and this is in agreement with other studies that show that synthetic H3 agonists have pain-relieving and anti-inflammatory effects [67]. A possible explanation for this discrepancy could be an internalization of the H3 receptors observed after a persistent pharmacological stimulation and, therefore, their consequent reduced activity/sensitivity [67, 68].

We found here that PEA-OXA repeated administration increased histamine levels in the hippocampal CA3 sub-region in sham mice. However, acute administration of PEA-OXA did not change the histamine release whereas transiently increased the levels of norepinephrine in sham mice (see Additional file 4: Figure S4).

We are unable here to reveal the exact mechanism through which PEA-OXA has produced this effect, however the action as an antagonist on alpha2 adrenergic receptors expressed on histaminergic terminals and serving as hetero-receptors could be, at least in part, at the base of the histamine increase. We can speculate that under physiological conditions PEA-OXA is acting mainly as alpha2 receptor antagonist, and that its agonist activity on histamine H3 component is instead masked. This would also depend on the different expression of the two receptors at the level of the hippocampal CA3 region, and it would be interesting to evaluate the effect of the PEA-OXA in other parts of the brain, such as cortical areas and the striatum, where H3 receptors are widely expressed [59].

In fact, histamine levels were also increased in the CA3 area of SNI mice chronically treated with the vehicle, possibly due to a compensatory mechanism that further highlights the complex brain modifications occurring in neuropathic pain. Indeed, neuropathic mice also showed increased levels of GABA that might, in turn, be responsible for inhibiting the release of other neurotransmitters such as catecholamines, which facilitate the histamine outflow by reducing alpha 2 heteroreceptors stimulation. Whatever the underlying mechanism, we found that PEA-OXA normalized the release of histamine, an effect that would be mediated by its partial agonist action on the H3 receptor acting as self-limiting histaminergic autoreceptor, together with an antagonism on the alpha2 receptors on histaminergic terminals. Moreover, PEA-OXA could behave as a protean agonist as recent reports highlighted this possibility also for the H3 receptors [69, 70]. The protean-like activity of PEA-OXA on the H3 receptors could explain, at least in part, some of the opposite effect of this compound on the histamine release in sham or neuropathic conditions. However, we observed that PEA-OXA modified the histamine levels only when administered chronically. Indeed, acute administration of PEA-OXA, at the same dosage, did not change the histamine level neither in sham nor in SNI mice. Therefore, the change in the neurotransmitters could be an effect mediated by both the H3 and alpha2 pharmacological modulation as well as adaptive processes induced by the drug in repeated administration regimen. In addition, we cannot exclude an involvement of the H2 histamine receptors in the PEA-OXA activity. Indeed, as elegantly suggested in a recent review by Obara [23] and coworkers, beside the well-established role of the H2 receptors in regulating proton pump at the parietal gastric cell level, these receptors can also have an important role in pain transmission and chronification [71]. In our hands, the sequence alignment between H3 and H2 receptors showed that, differently from H1 and H4, the arginine residue interacting with PEA-OXA in the H3 binding pocket is conserved in H2. This result prompted us to build a 3D-model of H2 receptor using the turkey beta1-adrenergic as template, rather than the muscarinic receptor adopted for H3, since the former was identified as the best template from blast search in PDB. However, for comparison purposes, a 3D model was also built using the same template as for H3 receptor. In both models, the arginine is involved in H-bonds reinforced by ionic interaction with a glutamate residue, not occurring in H3 receptor. Indeed, docking study carried out on both H2 homology models showed that PEA-OXA does not preferentially interact with this arginine residue, probably due to the presence of the above-mentioned glutamate residue. Moreover, the binding poses of PEA-OXA within the H2 binding site are more dispersed than in H3. Furthermore, differently from H3 receptor, no mutagenesis data have been so far reported showing a role of this arginine residue in binding/activation of H2 receptor. Taken together, these data do not support a role of PEA-OXA in H2 receptor activation. However, since such theoretical result remains to be experimentally validated, we decided to exclude these data from the present manuscript. Future studies will be aimed to investigate the pleiotropic mechanisms of this new molecule.

The complex dynamic profile of PEA-OXA on H3 receptor is also highlighted by its capability to partially counteract the hypokinetic effect induced by the potent H3 agonist immepip that we found in this study.

In addition to the increase in histamine levels, a decrease in DA and NA in the CA3 area of the hippocampus was also observed in SNI mice chronically treated with vehicle (see below).

### Effects of PEA-OXA on mechanical allodynia, dopamine and norepinephrine release in the hippocampus, and LC neural activity

In this study we observed that PEA-OXA, while on the one hand partially relieved allodynia in SNI mice, it induced a paradoxical allodynia in sham animals. This discrepancy may again reflect the different neuronal and receptor substrate on which PEA-OXA may act in the two conditions (healthy or pathological). Accordingly, previous evidence has shown that the effect of selective adrenergic alpha2 receptor antagonists on the pain response (i.e. allodynia) may change depending on the pain test used, as well as on the site of the drug administration (spinal or supra-spinal brain nuclei) [72–74]. The microinjection of an adrenergic alpha2 receptor antagonist in the LC was shown to exert anti-allodynic effects, which were prevented if the same antagonist was pre-administered at the spinal level. It is noteworthy that the same treatment performed in healthy animals facilitated thermal and mechanical nociception [74]. Therefore, it is likely that the opposite effects of the alpha2 adrenergic agonists or antagonists on pain thresholds may depend on both the type of pain condition and the anatomical site of receptor stimulation [73, 74].

SNI, which produced a significant decrease in the pain threshold, had no effect on tonic activity of LC neurons, as previously observed in other neuropathic pain models. What changed instead was the number of neurons found in control and SNI mice, which we demonstrated to be adrenergic through the systemic injection of medetomidine and idazoxan, an adrenergic alpha2 agonist and antagonist, respectively. However, chronic treatment with PEA-OXA significantly increased the tonic firing rate of LC neurons [75] in both SNI and sham mice. This effect is consistent with the action of PEA-OXA as an alpha2 adrenergic receptor antagonist, which normally inhibits adrenergic tone and when blocked by PEA-OXA produces an increase in extracellular NA levels and LC activity [51, 76]. Furthermore, PEA-OXA treatment induced an increase in the percentage of the spikes in burst, which is the main parameter indicating the enhancement of NA release [77].

Consistent with the cognitive and affective impairments observed in SNI mice, we found here that NA and DA levels were decreased in the CA3 area of these mice. The monoamine-induced priming of synaptic events facilitates neurons to properly adapt to an incoming input and finalize cell plasticity. Thus, the decreased levels of NA and DA levels in the CA3 area of SNI mice might explain in part why during conditions of long-term malaise, such as neuropathic pain or any other form of chronic stress, both depression-like signs and memory deficits can be observed. Accordingly, and consistent with its ameliorative effects on depression-like behaviors and cognitive performance, we found in the current study that PEA-OXA increased NA and DA levels in SNI mice. Indeed, a reduction of the time of immobility in the tail suspension and forced swim tests, and improvement of spatial and working memory in the Morris water maze and Y maze tests, were after the chronic treatment with PEA-OXA in SNI mice (with a milder effect in sham mice). PEA-OXA acute administration also increased the level of norepinephrine in sham mice (Additional file 1: Fig. S1).

### Effects of PEA-OXA on GABA and glutamate levels, LTP in the LEC-DG pathway and spatial memory

In recent decades, a widely shared task force for the study of the consequences/alterations of chronic pain on limbic structures has highlighted various psychiatric aspects that occur during the *chronification* of pain, such as learning and memory [1, 5, 7, 44, 78–80]. In a previous study, SNI has already been shown to induce depression-like behaviors and memory deficits in rats by compromising LTP in the hippocampal CA3-CA1 synapses [10]. It is also noteworthy that a prerequisite for an antidepressant effect consists mainly in the synaptic strengthening of the DG of the hippocampus, obtained by stimulating the perforating path from the LEC [81]. In this study, in order to evaluate the effect of PEA-OXA on neuropathic pain-related neuropsychiatric consequences, we resumed studying the possible functional alterations affecting the LEC-DG circuit of the hippocampus [7]. We confirm here that SNI influences the LEC-DG functional connectivity, i.e. that neuropathic pain adversely affects LTP and causes maladaptive changes in the DG glutamatergic synapses. Thus, we found consistency between cognitive deficits in the hippocampus-dependent cognitive tests (the Morris water maze and the Y maze that measure learning and spatial memory and working memory, respectively, [82]) and the impairment of LTP in the DG after tetanization of the LEC in SNI mice. The cellular and molecular mechanisms underlying LTP impairment associated with neuropathic pain are still unclear and largely attributable to maladaptive neuroplasticity linked to glutamate and/or GABA level changes, BDNF downregulation and many other neurochemical alterations [83, 84]. We suggest here that biogenic amines could also be implicated in the dysregulation of LTP in the hippocampus and the associated cognitive and affective impairments in SNI mice [8].

However, also the extracellular levels of glutamate and GABA were increased in the hippocampus of SNI mice in the current study, thus suggesting a dysregulation of the activity of the hippocampus. The increase in the extracellular levels of glutamate is in line with our previous study showing a similar effect in the DG of SNI mice [7] (and with other studies that have reported an increase in supraspinal glutamate levels after nerve injury [2, 15, 85, 86]. The levels of GABA were also found increased, possibly as a homeostatic mechanism to counteract the altered glutamatergic tone. It is interesting to note that, as we have already shown in the mPFC [30], PEA-OXA was capable of increasing the GABAergic tone in naïve or sham mice; an effect that can be associated with the antagonist action of PEA-OXA on the adrenergic alpha2 heteroreceptors. The normalization of GABA levels is closely associated with the effect of antidepressant drugs in individuals who respond to therapy, since chronic depression is associated with a reduction in GABA levels in limbic areas such as the prefrontal cortex [87]. Therefore, the ability of adrenergic alpha2 receptors to regulate GABA and glutamate release plays a relevant role in the pathophysiology of disorders that include affective, psychotic and cognitive symptoms. In this context, the non-selective blockade of adrenergic alpha2 receptors can play a fundamental role in the complex and not completely understood antidepressant action of molecules such as mirtazapine, mianserin, etc. or of antipsychotics such as clozapine. In agreement with these considerations, and as mentioned above, chronic administration with PEA-OXA improved the cognitive deficits in SNI mice, possibly by acting as an alpha2 receptor antagonist.

### Effects of PEA-OXA on sensory, emotional, and cognitive behaviour

The neurochemical and electrophysiological outcomes of the current study are consistent with the behavioural changes observed in SNI mice after PEA-OXA chronic treatment. Indeed, we have reported that, along with the partial recovery of LTP in the DG of SNI mice, and the normalization of extracellular glutamate, GABA, histamine, DA and NA levels in the hippocampus, PEA-OXA counteracted both depression-like behaviours and cognitive impairment. We cannot exclude that the analgesic properties of PEA-OXA may induce a “secondary” ameliorating action on the neurological SNI-mediated behavioral abnormalities, mostly associated with supraspinal mechanisms. However, because of the capability of PEA-OXA to modulate neurotransmitters release (also in physiological condition) in brain areas controlling not only pain, but also memory and reward, it is reasonable that PEA-OXA may play a role in different neuropsychological functions independently of pain control.

## Conclusions

The in vivo and in vitro data presented here collectively suggest a crucial mechanistic and functional link underlying the phenotypic changes in pathological conditions and the therapeutic effect of PEA-OXA in contrasting the central *sequelae* associated with SNI and the consequent chronic pain condition. The development of multi-target plant-based molecules, such as PEA-OXA, is now widely regarded as a promising therapeutic strategy for multidimensional neuro-psychiatric diseases, such as neuropathic pain.

## Supplementary Information

**Additional file 1: Figure S1**: Cell viability assay performed in histamine H3 and H4 receptors transfected COS cells treated with PEA-OXA. (A) Bar graph showing the cell viability assay measured in COS H3 (B) and H4 (C) cells using MTT assay. Each data represents the mean ± S.E.M. of four separate determinations.

**Additional file 2: Figure S2**: Effect of PEA-OXA in COS cells stably expressing human histamine H4 and H1 receptors. (A) Scatter plots showing the effect of PEA-OXA in COS cells expressing histamine H4 receptors on intracellular cAMP levels. (B) Bar graph showing the quantification of [Ca2 +]i measurements performed in histamine H1 receptor transfected COS cells. (C, D) Representative images showing the Ca^2+^ oscillations in COS H1 cells following the preincubation with PEA-OXA 5 µM and stimulation with histamine 100 µM and/or thapsigargin 5 µM. (D) Data represent the mean ± SEM of ≥ 5 determinations. Data sets were compared by use of one-way ANOVA followed by Bonferroni’s test. The asterisk denotes a p value ≤ 0.05.

**Additional file 3: Figure S3.** Effect of the chronic treatment with vehicle (kolliphor 5% in saline, v/v) or PEA-OXA (10 mg/kg) on anxiety-like behavior. “A” shows the effect of vehicle or PEA-OXA on the percentage of open arm-choice in the elevate plus-maze in sham and SNI mice. “B” and “C” show the effect of vehicle or PEA-OXA on the time spent in the dark box in seconds and the number of transitions in the light–dark box, respectively. “D” and “E” show the effect of vehicle or PEA-OXA on number of diggings and marble buried in the marble burying. Experiments have been carried out 30 days after SNI or sham surgery. Each point represents the mean ± S.E.M. Two-way ANOVA, followed by Tukey's post hoc test for multiple comparisons test were used for statistical analysis. p < 0.05 was considered statistically significant. Symbols indicate significant differences: *vs Sham/veh (p < 0.05), **vs Sham/veh (p < 0.01), ***vs Sham/veh (p < 0.001), ^#^vs SNI/veh (p < 0.05), respectively.

**Additional file 4: Figure S4.** Effect of a single intraperitoneal administration of vehicle (kolliphor 5% in saline, v/v) or PEA-OXA (10 mg/kg) on the extracellular levels of norepinephrine (NE) and (HA) in the hippocampus CA3 in sham and SNI mice. Experiments have been carried out 30 days after SNI or sham surgery. The black arrow indicates the administration of the vehicle or PEA-OXA. Each point represents the mean ± S.E.M of 8 animals per group. RM Two-way ANOVA, followed by Dunnett’s or Tukey's post hoc tests for multiple comparisons tests were performed for comparison vs BL and among groups during the time course, respectively. * indicates significant differences vs sham/veh (p < 0.05) and ^§^ indicates significant differences baseline (BL) (p < 0.05).

## Data Availability

The data and material are all available.
