# Correction to: 2-Pentadecyl-2-oxazoline ameliorates memory impairment and depression-like behaviour in neuropathic mice: possible role of adrenergic alpha2- and H3 histamine autoreceptors

**DOI:** 10.1186/s13041-021-00772-z

**Published:** 2021-04-01

**Authors:** Serena Boccella, Francesca Guida, Monica Iannotta, Fabio Arturo Iannotti, Rosmara Infantino, Flavia Ricciardi, Claudia Cristiano, Rosa Maria Vitale, Pietro Amodeo, Ida Marabese, Carmela Belardo, Vito de Novellis, Salvatore Paino, Enza Palazzo, Antonio Calignano, Vincenzo Di Marzo, Sabatino Maione, Livio Luongo

**Affiliations:** 1 Department of Experimental Medicine, Pharmacology Division, University of Campania “L. Vanvitelli”, 80138 Naples, Italy; 2 Department of Pharmacy, School of Medicine, University of Naples Federico II, Naples, Italy; 3 Institute of Biomolecular Chemistry, CNR, Pozzuoli, Italy; 4 Endocannabinoid Research Group, Institute of Biomolecular Chemistry, CNR, Pozzuoli, Italy; 5 Canada Excellence Research Chair on the Microbiome-Endocannabinoidome Axis in Metabolic Health, Université Laval, Quebec City, Canada; 6 IRCSS, Neuromed, Pozzilli, Italy

## Correction to: Mol Brain (2021) 14:28 https://doi.org/10.1186/s13041-020-00724-z

Following publication of the original article [1], the authors identified multiple errors throughout the article. The corrected errors are listed below and the original article has been updated to correct this.

## Abstract section

The second-to-last sentence of the Abstract was corrected. The updated sentence is given below and the changes have been highlighted in **bold typeface:**

Treatment for 14 days with PEA-OXA after the onset of the symptoms associated with neuropathic pain resulted in the following effects: (i) allodynia was decreased; (ii) affective/cognitive impairment associated with SNI (depression, spatial, and working memories) was counteracted; (iii) long-term potentiation in vivo in the lateral entorhinal cortex-dentate gyrus (perforant pathway, LPP) was ameliorated, (iv) hippocampal glutamate, GABA, histamine, **norepinephrine and dopamine altered levels after peripheral nerve injury were reversed**, (v) expression level of the TH positive neurons in the Locus Coeruleus were normalized.

## Figure 1 caption

The first few sentences of the caption for Fig. 1 were corrected. The updated caption is given below and the changes have been highlighted in **bold typeface:**

**Alpha2-mediated effect of PEA-OXA in vitro and in vivo. (a) Scatter plots showing the effect of PEA-OXA in COS cells stably expressing histamine H3 receptors on intracellular cAMP levels. Forskolin 10 uM served as AMPc inducer.** Data represent the mean ± S.E.M. of four separate determinations. Data sets were compared using t-Test and ANOVA followed by Tukey’s test. The asterisk indicates a p value ≤ 0.05 vs vehicle. The symbol (±) indicates a p value ≤ 0.05 vs forskolin. The symbol (°) indicates a p value ≤ 0.05 vs histamine. Effects of single injection of PEA-OXA (2.5 nmol/0.3 μl i.c.v.) on Immepip-induced decreased locomotor activity in into third ventricle (I3V) of Naϊve mice (**b**, **c**). Representation of coronal sections of the mouse brain with the cannula placement in I3V (**b**), Representative traces of mouse movement during an open field test (**c** upper panel). Total distance traveled in OFT (**c** lower panel). Data are represented as mean ± SEM of 5 mice per group. **p < 0.01 and ^###^p < 0.001 indicate significant differences compared to ACSF or Immepip. One-Way ANOVA, followed by Holm-Sidak’s post hoc test for multiple comparisons test was performed. Effect of the chronic treatment with vehicle (kolliphor 5% in saline, v/v, i.p.) or PEA-OXA (10 mg/kg, i.p.) on the Histamine release in the hippocampus CA3 (**d**). Data are represented as mean ± SEM of 8 mice per group. Two-way ANOVA, followed by Tukey's post hoc test for multiple comparisons test were used for statistical analysis. p < 0.05 was considered statistically significant. Symbols indicate significant differences: ***vs Sham/veh (p < 0.0001) and ^##^vs SNI/veh (p < 0.001), respectively.

## Figures 2 and 3

Figures 2 and 3 were interchanged. The captions for these figures were captured correctly, but the Figures itself were interchanged. The correct figures and captions have been included.

**Fig. 2:**
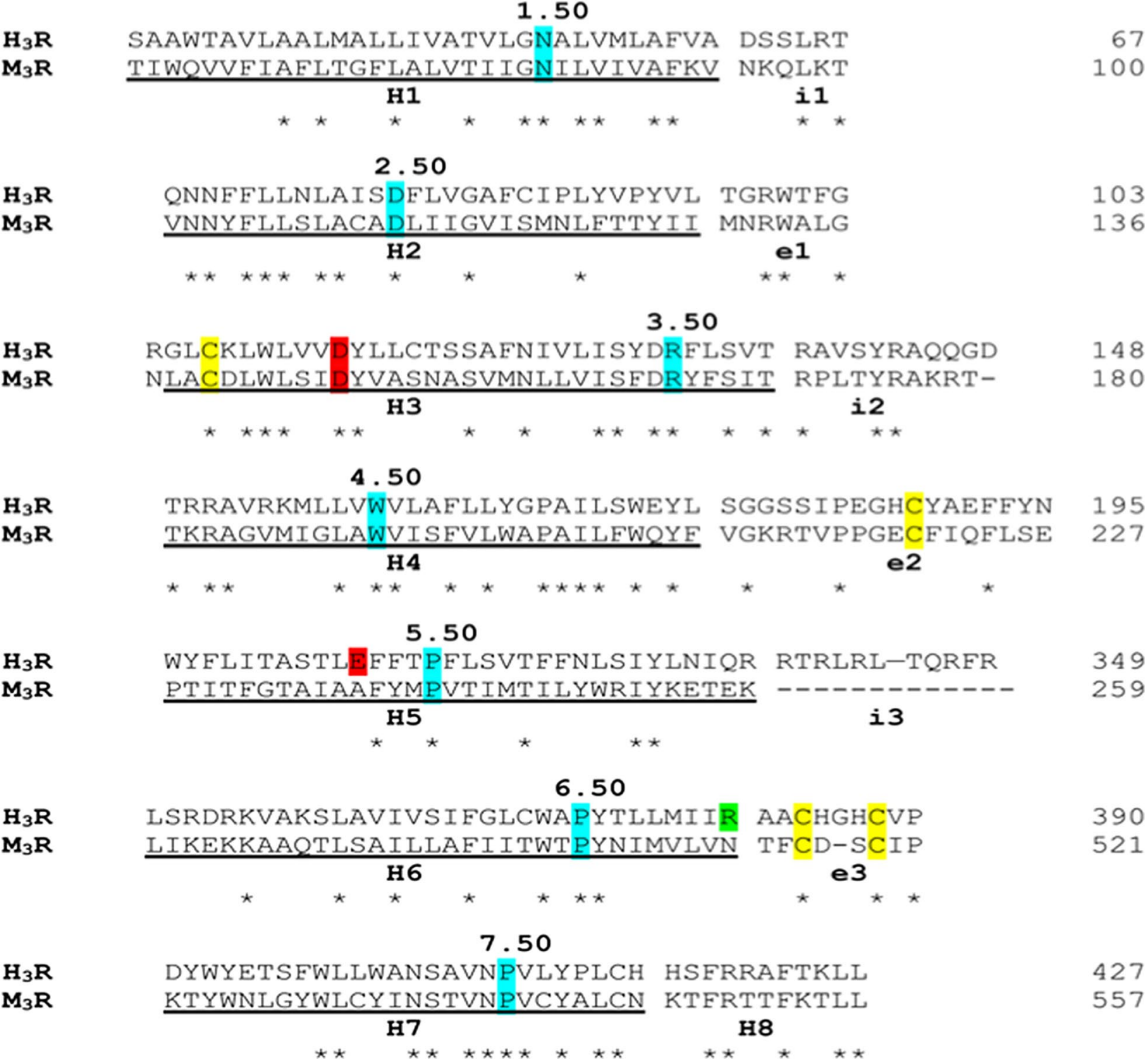
Structural alignment used for model building. Ballesteros–Weinstein numbers are used to label the most conserved residues within transmembrane helices, highlighted in cyan. Conserved cysteine residues involved in disulfide bridge are colored in yellow. The negatively charged residues Asp144 and Glu206 critical for histamine binding are colored in red while Arg381, proposed as relevant for PEA-OXA binding is colored in green. The long loop i3 was truncated in the model, leaving only the residues allowing a proper linker between the helices. The transmembrane helices H1–H8 are underlined while the intracellular and extracellular loops are labeled “i1–3” and “e1–3”, respectively

**Fig. 3:**
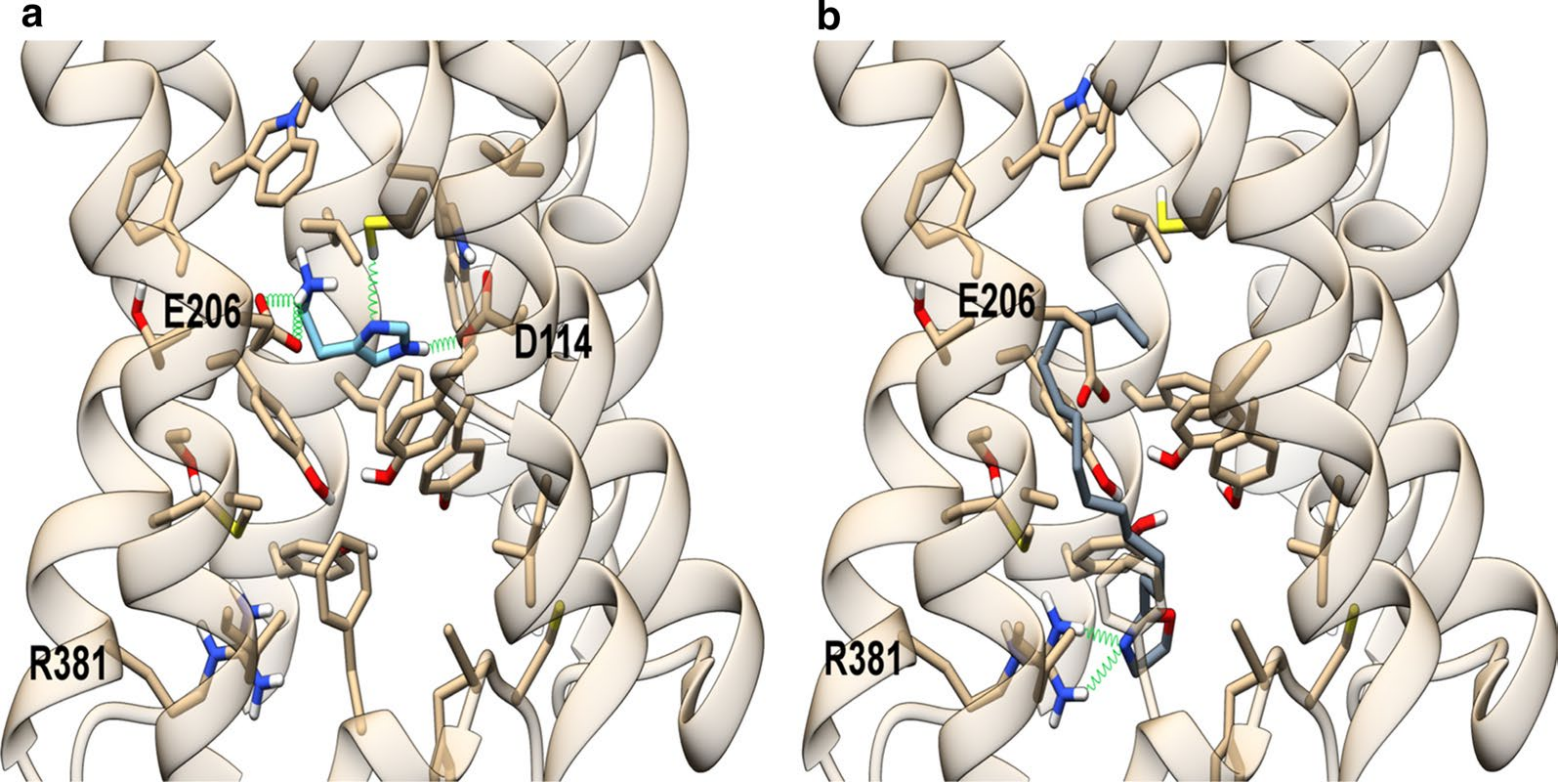
Energy minimized theoretical complexes of histamine H3 receptor with histamine (**a**) and PEA-OXA (**b**). Protein is colored in tan, histamine in light blue and PEA-OXA in slate gray. Ligands and residues within 5 Å from the ligands are shown in stick representation. Oxygen, sulfur and nitrogen heteroatoms are colored in red, yellow and blue, respectively. H-bonds are represented as green springs

## Theoretical complexes of the human histamine H3 receptor with histamine and PEA-OXA section

In the second-to-last sentence of the ‘Theoretical complexes of the human histamine H3 receptor with histamine and PEA-OXA’ section, the citation to Fig. 3 was removed.

## PEA-OXA pharmacological modulation of histamine H3 receptor: in vitro and in vivo evidence section

In the last sentence of the first paragraph of the ‘PEA-OXA pharmacological modulation of histamine H3 receptor: in vitro and in vivo evidence’ section, the word ‘positive’ was removed before ‘allosteric modulator’. The corrected sentence reads:

We cannot exclude that PEA-OXA might behave as a allosteric modulator of the H3 receptors as also suggested by the docking data.
