# Supplementary figures and images for: 2-Pentadecyl-2-oxazoline ameliorates memory impairment and depression-like behaviour in neuropathic mice: possible role of adrenergic alpha2- and H3 histamine autoreceptors
Source: Mol Brain. 2021 Feb 8;14:28. doi: 10.1186/s13041-020-00724-z (PMC7871413; doi:10.1186/s13041-020-00724-z)

**A**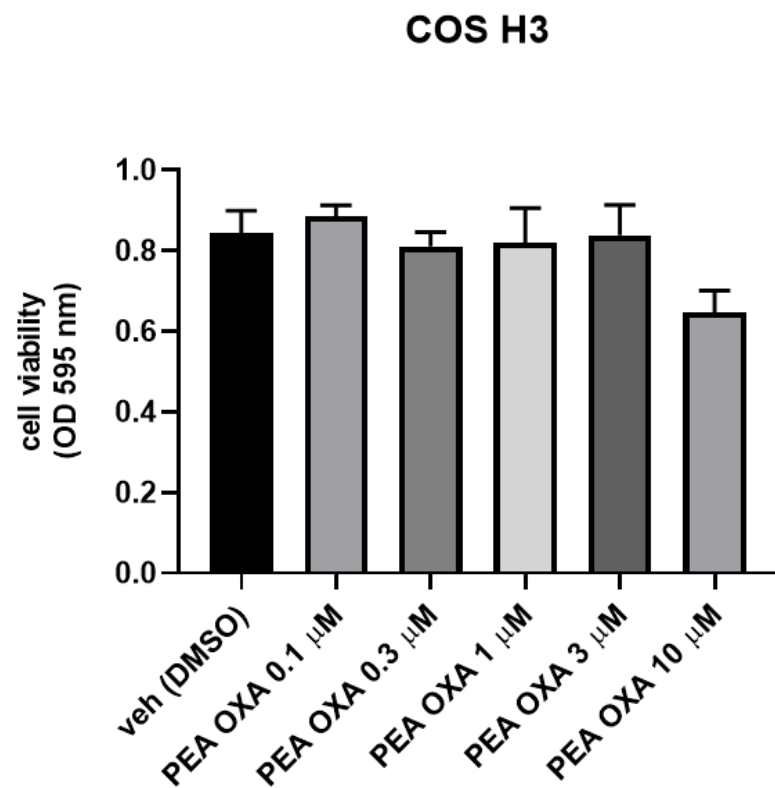**B**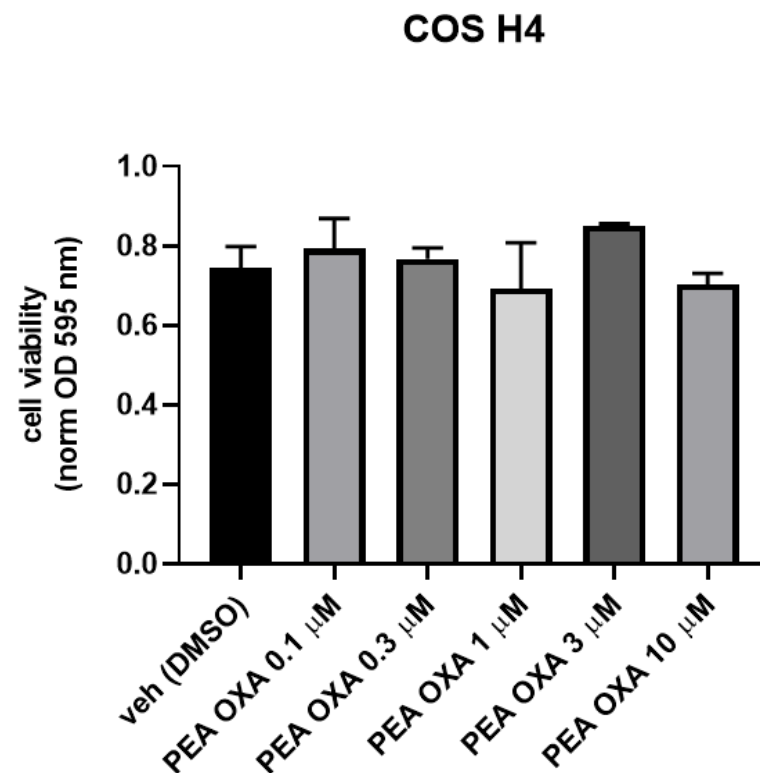

Supplement: Supplementary file 1 — Additional file 1: Figure S1: Cell viability assay performed in histamine H3 and H4 receptors transfected COS cells treated with PEA-OXA. (A) Bar graph showing the cell viability assay measured in COS H3 (B) and H4 (C) cells using MTT assay. Each data represents the mean ± S.E.M. of four separate determinations. [file 13041_2020_724_MOESM1_ESM.pdf]

**A**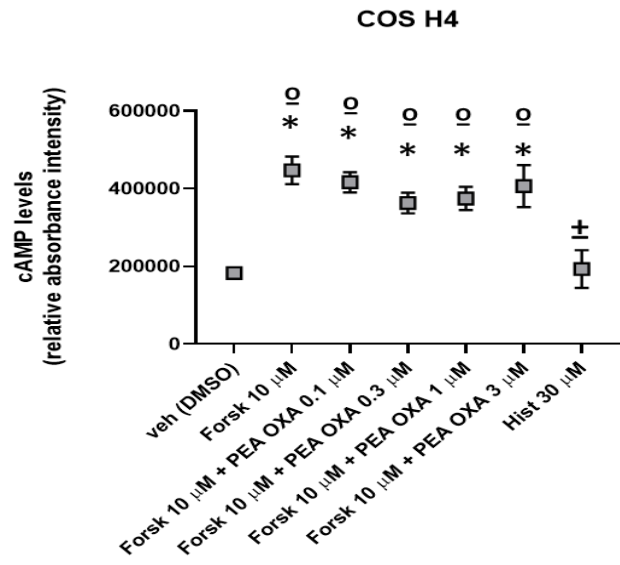**B**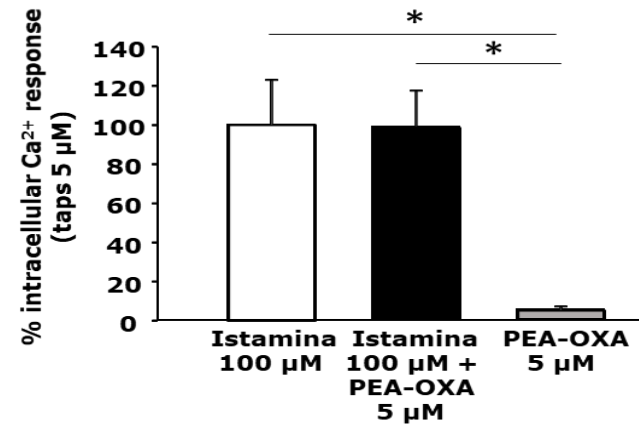**C**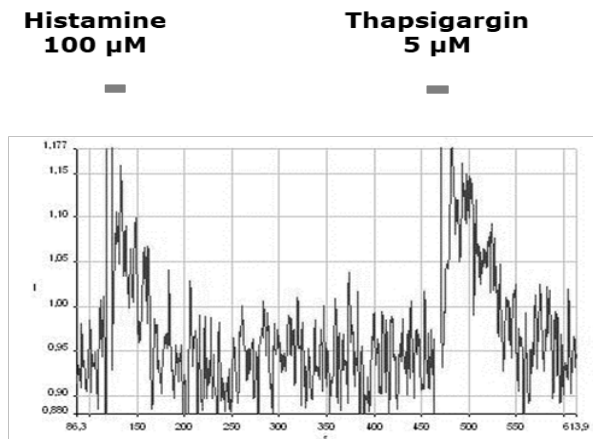**D**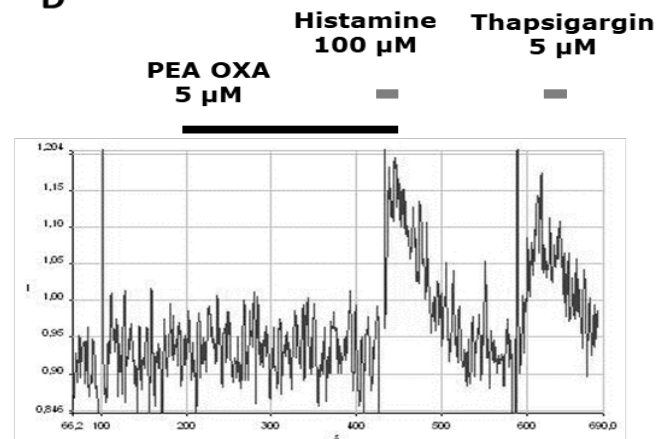

Supplement: Supplementary file 2 — Additional file 2: Figure S2: Effect of PEA-OXA in COS cells stably expressing human histamine H4 and H1 receptors. (A) Scatter plots showing the effect of PEA-OXA in COS cells expressing histamine H4 receptors on intracellular cAMP levels. (B) Bar graph showing the quantification of [Ca2 +]i measurements performed in histamine H1 receptor transfected COS cells. (C, D) Representative images showing the Ca2+ oscillations in COS H1 cells following the preincubation with PEA-OXA 5 µM and stimulation with histamine 100 µM and/or thapsigargin 5 µM. (D) Data represent the mean ± SEM of ≥ 5 determinations. Data sets were compared by use of one-way ANOVA followed by Bonferroni’s test. The asterisk denotes a p value ≤ 0.05. [file 13041_2020_724_MOESM2_ESM.pdf]

**A**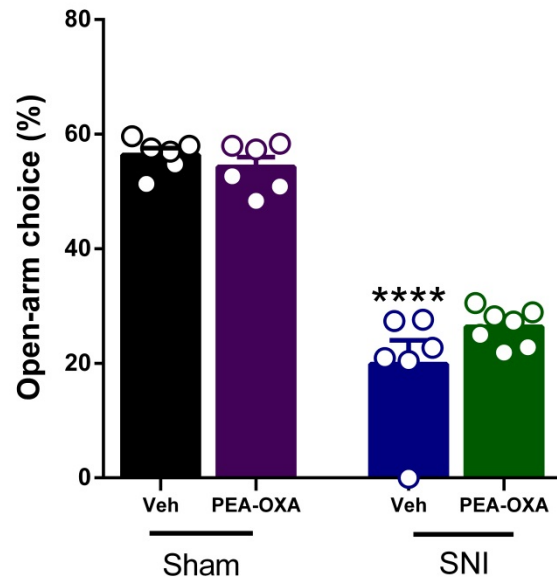**B**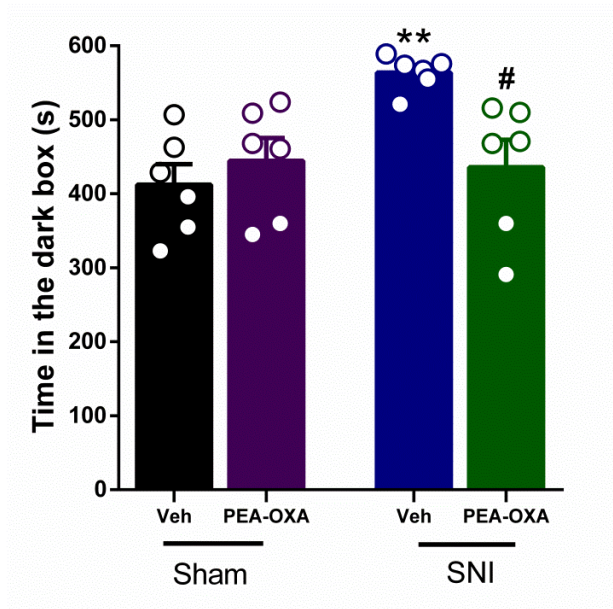**C**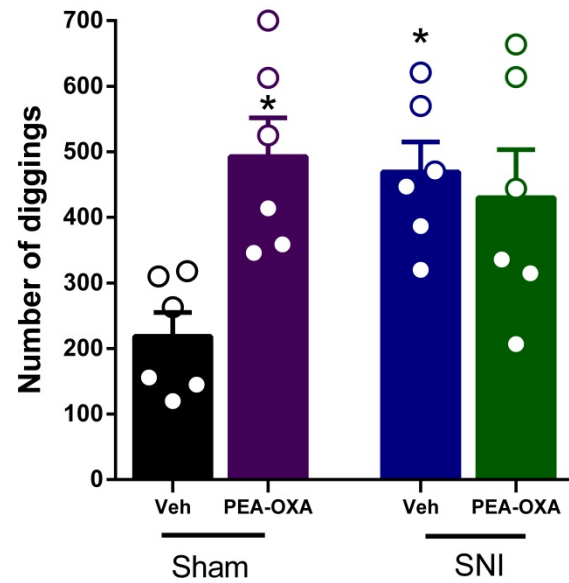**D**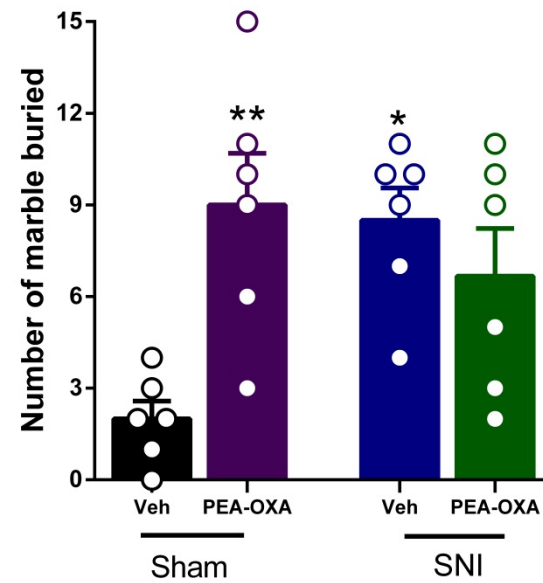

Supplement: Supplementary file 3 — Additional file 3: Figure S3. Effect of the chronic treatment with vehicle (kolliphor 5% in saline, v/v) or PEA-OXA (10 mg/kg) on anxiety-like behavior. “A” shows the effect of vehicle or PEA-OXA on the percentage of open arm-choice in the elevate plus-maze in sham and SNI mice. “B” and “C” show the effect of vehicle or PEA-OXA on the time spent in the dark box in seconds and the number of transitions in the light–dark box, respectively. “D” and “E” show the effect of vehicle or PEA-OXA on number of diggings and marble buried in the marble burying. Experiments have been carried out 30 days after SNI or sham surgery. Each point represents the mean ± S.E.M. Two-way ANOVA, followed by Tukey's post hoc test for multiple comparisons test were used for statistical analysis. p < 0.05 was considered statistically significant. Symbols indicate significant differences: *vs Sham/veh (p < 0.05), **vs Sham/veh (p < 0.01), ***vs Sham/veh (p < 0.001), #vs SNI/veh (p < 0.05), respectively. [file 13041_2020_724_MOESM3_ESM.pdf]

## Slide 1
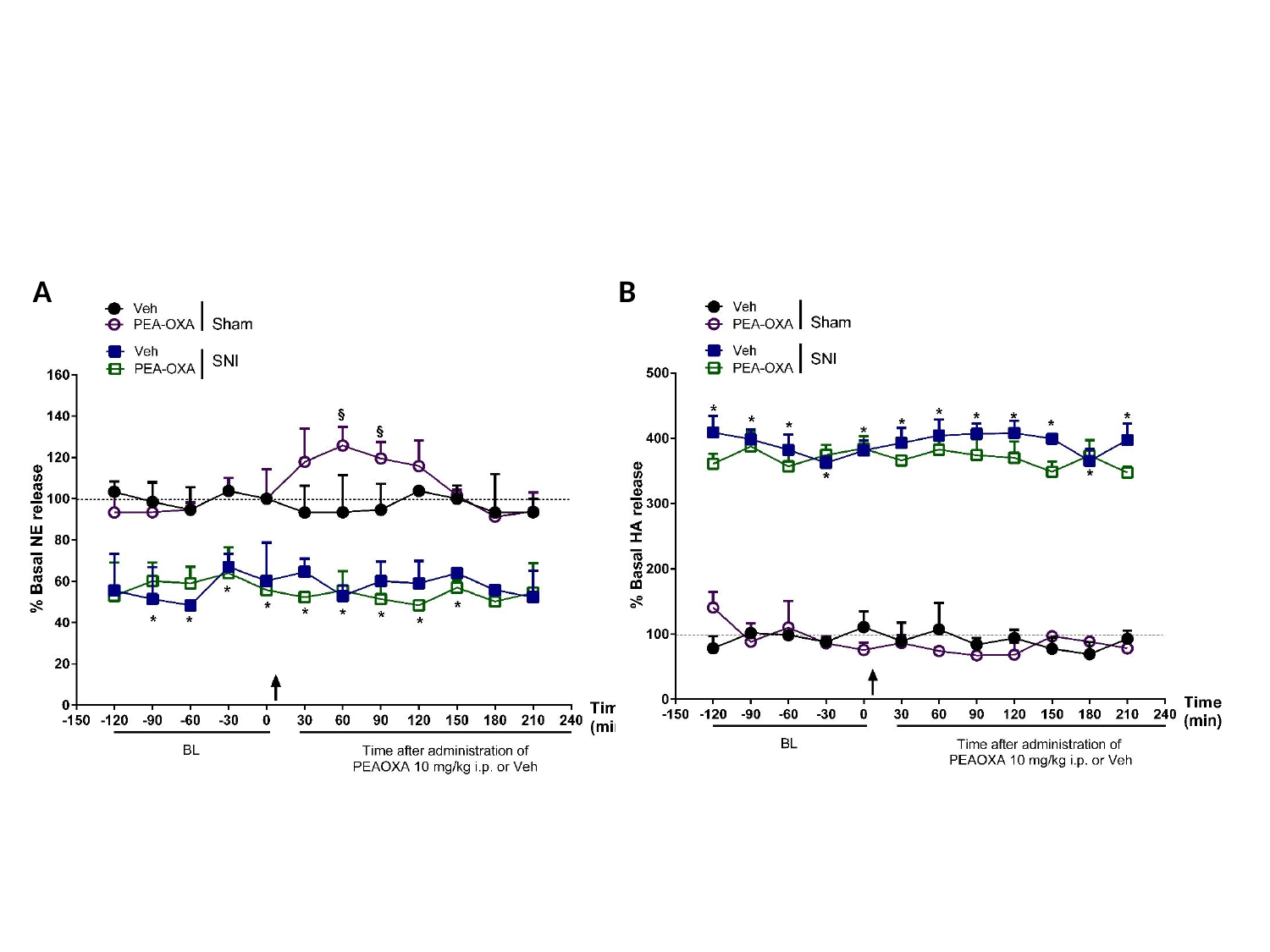

A
B

Supplement: Supplementary file 4 — Additional file 4: Figure S4. Effect of a single intraperitoneal administration of vehicle (kolliphor 5% in saline, v/v) or PEA-OXA (10 mg/kg) on the extracellular levels of norepinephrine (NE) and (HA) in the hippocampus CA3 in sham and SNI mice. Experiments have been carried out 30 days after SNI or sham surgery. The black arrow indicates the administration of the vehicle or PEA-OXA. Each point represents the mean ± S.E.M of 8 animals per group. RM Two-way ANOVA, followed by Dunnett’s or Tukey's post hoc tests for multiple comparisons tests were performed for comparison vs BL and among groups during the time course, respectively. * indicates significant differences vs sham/veh (p < 0.05) and § indicates significant differences baseline (BL) (p < 0.05). [file 13041_2020_724_MOESM4_ESM.pptx]
